# Immunity-and-matrix-regulatory cells enhance cartilage regeneration for meniscus injuries: a phase I dose-escalation trial

**DOI:** 10.1038/s41392-023-01670-7

**Published:** 2023-11-01

**Authors:** Liangjiang Huang, Song Zhang, Jun Wu, Baojie Guo, Tingting Gao, Sayed Zulfiqar Ali Shah, Bo Huang, Yajie Li, Bo Zhu, Jiaqi Fan, Liu Wang, Yani Xiao, Wenjing Liu, Yao Tian, Zhengyu Fang, Yingying Lv, Lingfeng Xie, Sheng Yao, Gaotan Ke, Xiaolin Huang, Ying Huang, Yujuan Li, Yi Jia, Zhongwen Li, Guihai Feng, Yan Huo, Wei Li, Qi Zhou, Jie Hao, Baoyang Hu, Hong Chen

**Affiliations:** 1 Department of Rehabilitation, Tongji Hospital, Tongji Medical College, Huazhong University of Science and Technology, Wuhan, China; 2 National Stem Cell Resource Center, State Key Laboratory of Stem Cell and Reproductive Biology, Institute of Zoology, Institute for Stem Cell and Regeneration, Chinese Academy of Sciences, Beijing, China; 3 Beijing Institute for Stem Cell and Regenerative Medicine, Beijing, China; 4 Department of Radiology, Tongji Hospital, Tongji Medical College, Huazhong University of Science and Technology, Wuhan, China; 5 Stem Cell Research Center, Tongji Hospital, Tongji Medical College, Huazhong University of Science and Technology, Wuhan, China; 6 Department of Orthopedics, Tongji Hospital, Tongji Medical College, Huazhong University of Science and Technology, Wuhan, China; 7 University of Chinese Academy of Sciences, Beijing, China; 8 Beijing Key Lab for Pre-clinical Safety Evaluation of Drugs, National Center for Safety Evaluation of Drugs, National Institutes for Food and Drug Control, Beijing, China; 9 Beijing Zephyrm Biotechnologies Co., Ltd., Beijing, China

**Keywords:** Stem-cell research, Translational research

## Abstract

Immunity-and-matrix-regulatory cells (IMRCs) derived from human embryonic stem cells have unique abilities in modulating immunity and regulating the extracellular matrix, which could be mass-produced with stable biological properties. Despite resemblance to mesenchymal stem cells (MSCs) in terms of self-renew and tri-lineage differentiation, the ability of IMRCs to repair the meniscus and the underlying mechanism remains undetermined. Here, we showed that IMRCs demonstrated stronger immunomodulatory and pro-regenerative potential than umbilical cord MSCs when stimulated by synovial fluid from patients with meniscus injury. Following injection into the knees of rabbits with meniscal injury, IMRCs enhanced endogenous fibrocartilage regeneration. In the dose-escalating phase I clinical trial (NCT03839238) with eighteen patients recruited, we found that intra-articular IMRCs injection in patients was safe over 12 months post-grafting. Furthermore, the effective results of magnetic resonance imaging (MRI) of meniscus repair and knee functional scores suggested that 5 × 10^7^ cells are optimal for meniscus injury treatment. In summary, we present the first report of a phase I clinical trial using IMRCs to treat meniscus injury. Our results demonstrated that intra-articular injection of IMRCs is a safe and effective therapy by providing a permissive niche for cartilage regeneration.

## Introduction

Meniscus injuries are the second most commonly occurring injuries to the knee.^1^ Due to poor vascularization, especially in the inner two-thirds of the avascular zone, meniscus tissue’s ability to heal following damage is minimal.^2,3^ The main treatments for meniscus injuries, including physiotherapy, pharmacological interventions and glucocorticoid injections, are unsatisfactory.^4^ Most patients end up with orthopedic surgeries,^5,6^ but the long-term prognosis of patients with meniscectomy surgeries remains questionable due to concerns of osteoarthritis (OA).^7,8^ It is hence increasingly considered that meniscus tissue should be repaired and retained as much as possible instead of resection.^9^ So far, no FDA-approved drugs are genuinely effective in repairing the injured meniscus. Therefore, an unmet need exists to develop innovative therapeutic strategies for articular meniscus repair.

Stem cell-based regenerative therapy is an emerging and promising option for healing meniscus injury.^10^ Mesenchymal stem cells (MSCs) are multipotent cells with a self-renewal and differentiation ability for cartilage tissues.^11^ MSCs exert tissue repair functions through their biological properties, including low immunogenicity,^12^ differentiation capacity to chondrocytes,^13^ and undefined paracrine mediators such as endothelial cytokines, epithelial growth factor (EGF), anti-inflammatory cytokines, and antimicrobial peptides.^14^ One possible mechanism by which MSCs repair the damaged meniscus may be related to the potential of MSCs to differentiate into chondrocytes^15^ and fibrous matrix.^16^ Another even more critical way is to secrete a series of cytokines, promoting meniscal healing via paracrine pathways.^17^ However, the cytokines and the corresponding signaling pathways that regulate meniscus regeneration remain unclear.^18^

To date, one double-blinded randomized controlled trial^19^ and a few cases reports^20–25^ evaluated the potential of allogeneic MSCs in treating meniscus injuries. Those clinical studies provided hints about the beneficial effects of primary MSCs on meniscus regeneration via MRI, knee pain relief, and improvement in the range of knee movement.^19–21^ Nonetheless, primary MSCs employed in these clinical trials originate from sources like the umbilical cord, bone marrow, or adipose tissue. However, they exhibit limitations such as donor and tissue heterogeneity, inadequate quality control during cell production, and restricted self-renewal potential and lifespan, impeding their broader clinical application. In contrast, IMRCs, derived from clinical-grade human embryonic stem cells (hESCs) following strict quality standards (GMP), exhibit typical MSC-like features.^26,27^ These IMRCs, also known as immunity- and matrix-regulatory cells, possess unique capabilities for immune regulation and matrix production, surpassing umbilical cord mesenchymal stem cells (UCMSCs). They not only maintain regular MSCs characteristics but also show functional stability for quality control, enabling large-scale GMP-compliant production. Prior research has demonstrated their potential in immunomodulation and tissue repair. For instance, the injection of IMRCs has the capability to suppress pulmonary inflammation and fibrosis following acute lung injury in vivo,^26,28^ improve cognitive ability in early-stage AD mice,^29^ prevent chronic cerebral hypoperfusion induced white matter injury and cognitive impairment,^30^ ameliorate the progression of osteoarthritis,^31^ etc. Meanwhile, IMRCs-derived extracellular vesicles and secretomes can attenuate pulmonary fibrosis.^32,33^ Nevertheless, it is not known if the IMRCs have a similar therapeutic effect in meniscus-injured patients.

In the present study, we found that hESCs-derived-IMRCs present strong immunomodulatory and pro-regenerative profiles when stimulated by synovial fluids from patients with meniscus injury. Endogenous fibrocartilage regeneration was observed in meniscal defect rabbits after IMRCs engraft. Following additional safety assays in cynomolgus monkeys, we conducted a phase I, dose-escalation clinical study (NCT03839238) and found that intra-articular injection of IMRCs in patients is safe with promising outcomes for meniscus injuries. To our knowledge, this is the first-in-human Phase I clinical study of IMRCs in meniscus injuries.

## Results

### IMRCs possess a stronger immunomodulatory and pro-regenerative profile than UCMSCs

**Fig. 1:**
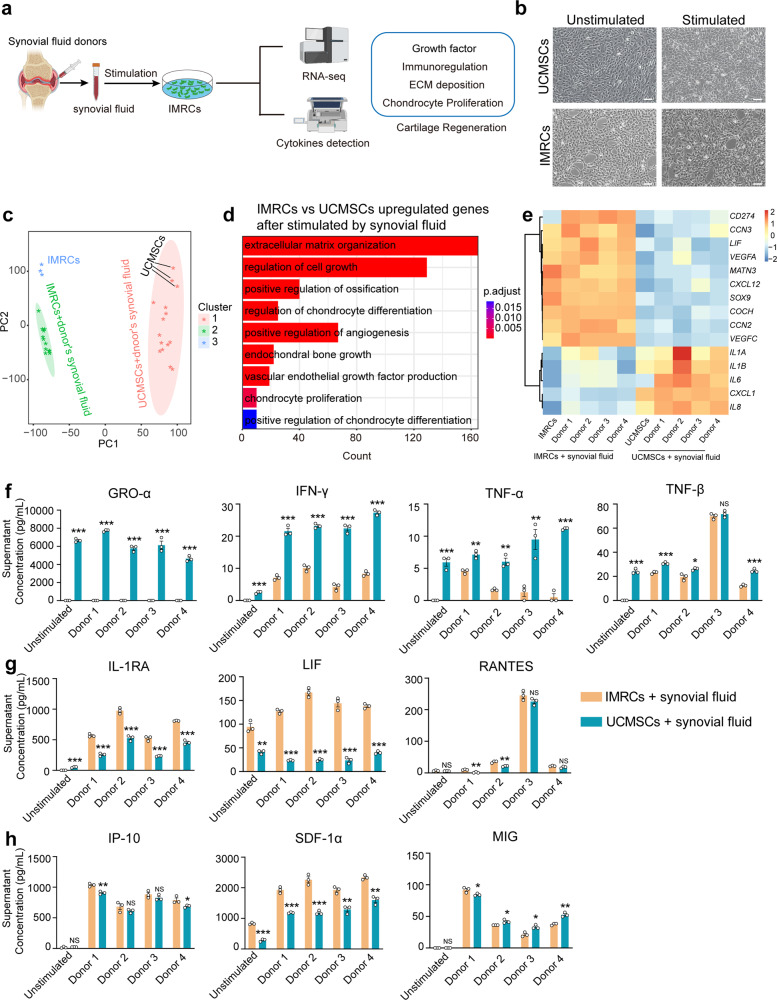
IMRCs activated by synovial fluid enhanced chondrocytes proliferation and differentiation. **a** Schematic illustration of the cell model stimulated by synovial fluid in vitro. **b** Morphology of UCMSCs and IMRCs before and after synovial fluid stimulation. **c** PCA of gene expression values derived from whole transcriptome sequencing data of IMRCs and UCMSCs before and after synovial fluid treatment. **d** GO biological process (GOBP) analysis of differentially upregulated genes for IMRCs versus UCMSCs after being treated by synovial fluid. **e** Heatmap illustrating the gene expression in UCMSCs and IMRCs, before and after synovial fluid stimulation. ELISA analysis of biologically relevant chemokines and cytokines in the supernatant of IMRCs and UCMSCs with and without synovial fluid stimulation. Proinflammatory (**f**), immunomodulatory (**g**), and pro-regenerative (**h**), cytokines. *P < 0.05, **P < 0.01, ***P < 0.001, NS, not significant; data are represented as the mean ± SEM. Scale bar: 100 μm

In this study, IMRCs derived from hESCs were generated through the passage of migrating cells acquired from human embryoid bodies (hEBs) using serum-free reagents (Supplementary Fig. 1a).^26^ IMRCs expressed MSC-specific markers, which included CD29, CD73, CD90, and CD105 (Supplementary Fig. 1b). Furthermore, IMRCs exhibited a remarkable capacity for tri-lineage differentiation into mesenchymal tissues, including adipocytes, chondroblasts, and osteoblasts (Supplementary Fig. 1c). Additionally, IMRCs displayed the ability to inhibit PBMC proliferation (Supplementary Fig. 1d). To explore the regenerative and immunomodulatory potential of IMRCs, we used the synovial fluids from four donors with meniscus injuries (Supplementary Table 1) to mimic the actual micro-environment in the knee joint cavity and established a cell model for in vitro assessment and mechanism investigation (Fig. 1a). After stimulation with synovial fluid, the IMRCs displayed distinct morphological changes including spindle-shaped soma and elongated cell body (Fig. 1b). At the transcriptomic level, principal component analysis (PCA) showed that IMRCs, rather than UCMSCs, presented a significantly different cluster of cells exhibiting distinct cellular properties after co-culture with synovial fluid (Fig. 1c). The global gene analysis reveals that, following synovial fluid stimulation, upregulated genes in IMRCs are enriched in pathways related to mesenchymal cell proliferation, regulation of cartilage development, and cell growth, among others (Supplementary Fig. 2a). Highly expressed genes in stimulated IMRCs were enriched with chondrocyte proliferation and vascular endothelial growth factor production, whereas genes more highly expressed in UCMSCs were clustered in inflammation-related pathways (Fig. 1d, Supplementary Fig. 2b and Supplementary Table 2). Consequently, we observed that some pro-inflammatory genes exhibited lower expression levels in IMRCs compared to UCMSCs, while some extracellular matrix-associated genes and growth factors, such as *SOX9*, which was proven to be an essential regulator of cartilage regeneration, were expressed at higher levels in IMRCs (Fig. 1e). In addition, we co-cultured rat chondrocytes with an IMRCs-conditioned medium and found that the conditioned medium can promote the migration of rat chondrocytes (Supplementary Fig. 3). Whole-transcriptome analysis confirmed that chondrocytes and S-chondrocytes clustered separately in an unsupervised hierarchical clustering (Supplementary Fig. 3a). Global gene analysis revealed that highly expressed genes in S-chondrocytes were enriched with pathways related to bone mineralization (Supplementary Fig. 3b). Moreover, KEGG analysis showed that the upregulated DEGs between chondrocytes and S-chondrocytes groups were enriched in 5 pathways, including the HIF-1 signaling pathway, MAPK signaling pathway and mineral absorption pathway (Supplementary Fig. 3c). We also found that the IMRCs-conditioned medium can promote the migration of chondrocytes. Meanwhile, we found a significant upregulation of *SOX9* expression in the S-chondrocytes group (Supplementary Fig. 3d). In chondrocytes, *SOX9* is a key factor in maintaining the characteristics and functionality of cartilage tissue.^34–36^ It is involved in regulating the proliferation, differentiation, and matrix synthesis of chondrocytes. We also found that the IMRCs-conditioned medium can promote the migration of chondrocytes (Supplementary Fig. 3e, f).

In order to obtain a more comprehensive understanding of IMRCs at the protein level, we conducted a focused ELISA analysis involving 48 biologically relevant chemokines and cytokines in response to synovial fluid stimulation (Supplementary Table 3). The results indicated that upon synovial fluid stimulation, UCMSCs exhibited high expression levels of pro-inflammation cytokines, including GRO-α, IFN-γ, TNF-α, TNF-β, and IL-8 (Fig. 1f and Supplementary Fig. 4), In contrast, both before and after stimulation, IMRCs displayed elevated expression of anti-inflammatory cytokines IL-1RA, and the immunomodulatory cytokines LIF, RANTES (Fig. 1g). Additionally, among the pro-regenerative cytokines, we observed increased levels of SDF-1α, IP-10, MIG, PDGF-BB after stimulation (Fig. 1h and Supplementary Fig. 4). Notably, the SDF-1α and PDGF-BB played critical roles in cartilage matrix formation and cartilage defects repair.^37–39^ These results suggest that IMRCs possess a stronger immunomodulatory and cartilage pro-regenerative potential than UCMSCs when exposed to synovial fluid from patients with meniscus injuries.

### IMRCs have a good safety profile for administration in vivo

**Fig. 2:**
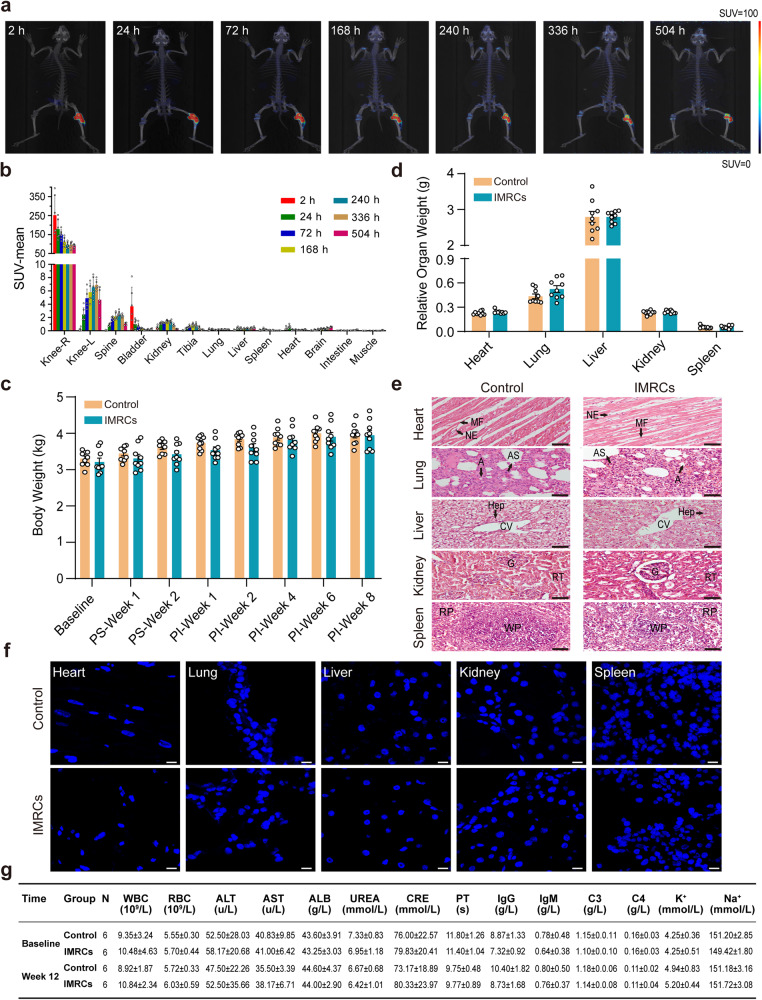
Toxicity test after cell transplantation. **a** The radioactive substances of representative rabbits are shown at several time points after right knee intra-articular injection. **b** Statistical plots of SUV-mean values of organs of rabbits at seven time points post-injection. Plot bar, mean ± SEM; n = 6 rabbits for each time point. **c** Changes in rabbit body weight at different time points throughout the experiment (n = 9). PS, post-surgery. PI, post-injection. **d** The weight of the internal organs of the rabbits at week 8 (n = 9). **c**, **d** Two-tailed independent sample t-test, **e** Representative images of HE staining of rabbits’ internal organs show no difference in the structure of internal organs between the two groups (n = 7 in each group). Micrographs of the hearts showed normal cardiac myocytes with regular conformation in the myocardial fibers (MF) and nucleus of the endothelium (NE). Histology of the lungs showed normal lung parenchyma with preserved structures of alveoli (A), alveolar sacs (AS). Histology of the liver sample showed normal morpho-functional units of the liver. Hepatocytes (Hep) were intact. Portal tracks converged around the lobular central vein (CV). The kidney showed a complete structure with a regularly shaped glomerulus (G), normal renal tubules (RT). The spleen was also morphologically normal with an intact cellular layout with regular sinusoidal spaces and prominent red pulp (RP) and white pulp (WP). Scale bar: 50μm. **f** Confocal images of FISH analysis at week 8 for detection of human cells in internal organs. Scale bar: 10 μm. **g** The long-term toxicity test in cynomolgus monkeys: blood test at baseline and week 12. IMRCs group: 2 × 10^7^ cells/kg. Data are presented as mean ± SEM

**Fig. 3:**
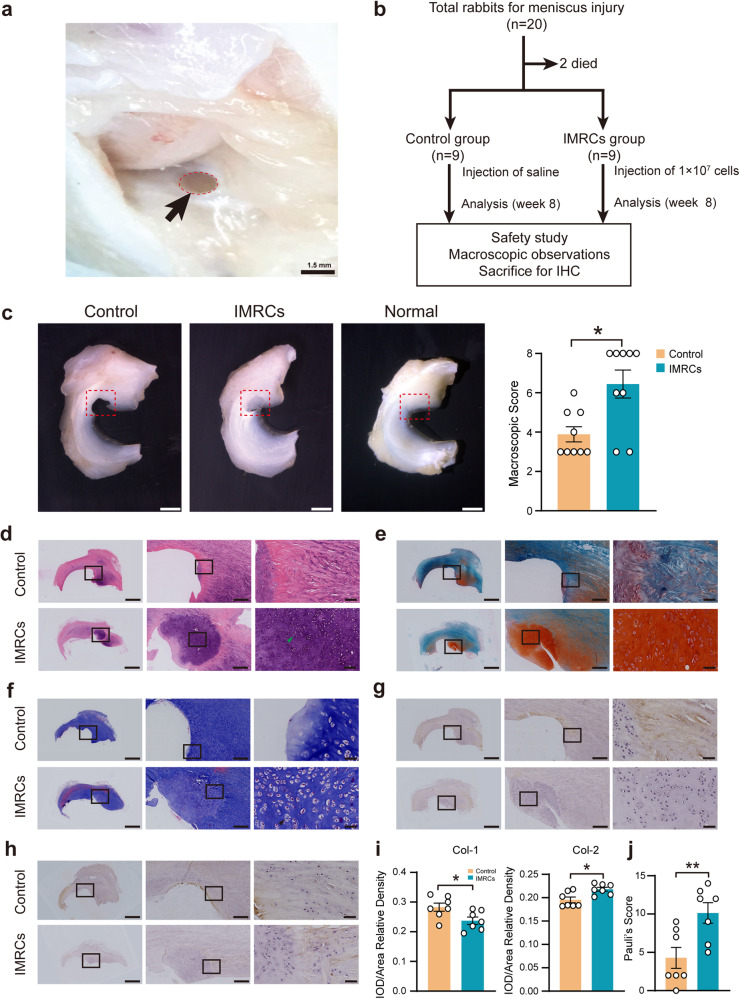
The study design, macroscopic observation, histological analysis and representative immunohistochemical staining 8 weeks after cell transplantation. **a** Rabbit knee joint meniscus defect model. A cylindrical defect with a diameter of 1.5 mm was formed at the anterior corner of the medial meniscus. Black arrows indicate the defect. **b** Experimental design and schematic diagram of animal distribution in each group. Dead rabbits were excluded from any analysis. IHC, immunohistochemistry. **c** The macroscopic images of meniscus repair after injury at week 8. Scale bar: 2 mm. And macroscopic score for the general view of meniscus repair at week 8, n = 9 in each group. *P* values, two-tailed Mann-Whitney tests. **d** HE staining of meniscus repair at week 8. Green arrowhead shows mature chondroid cells. Scale bar: 2.5 mm, 500 μm, 100 μm. **e** Safranin-O staining (SO). **f** Masson staining. Black arrows show chondrocytes in isogenous group. **g** Type I collagen (Col-1). **h** Type II collagen (Col-2). For (**e**)–(**h**), scale bar: 2.5 mm, 500 μm, 50 μm. The boxed areas are shown at higher magnification. **i** Semi-quantitative analyses of immunohistochemical staining for Col-1 and Col-2. *P* values, two-tailed independent sample t-test. **j** shows the result of Pauli’s score at week 8, n = 7 in each group. *P* values, two-tailed independent sample t-test. *P < 0.05, **P < 0.01; Data are presented as mean ± SEM

The IMRCs used in this study were the same batch as before, and the detailed karyotyping and in vivo tumorigenicity assays had been described in our previous article.^26^ To fully evaluate the safety of IMRCs, we performed a systematic evaluation using two different species of animals, rabbits and cynomolgus monkeys (*Macaca fascicularis*). IMRCs were radiolabeled with 89Zr-oxine complex for visualization with microPET/CT for evaluating the in vivo biodistribution. At 2, 24, 72, 168, 240, 336, and 504 h after intra-articular injection of rabbit, the radioactive label was retained mainly in the right knee joint cavity and rarely detected in other tissues or organs (Fig. 2a). The histogram of mean standard uptake value (SUV) gradually decreased to a steady level by 240 h (Fig. 2b). Then, we established a rabbit model of meniscus injury by punching a hole on the medial side of the right meniscus to form a 1.5 mm circular defect (Fig. 3a). Then the rabbits were randomized into the control and IMRCs groups (Fig. 3b). Intra-articular injection of a dose of 1 × 10^7^ IMRCs was performed in the IMRCs group. Throughout the whole experiment, the rabbits were injected with the immunosuppressant FK-506. The body weight and the relative organ weight showed no statistically significant difference between the two groups (Figs. 2c, d). Histological analysis of the IMRCs group showed a similar tissue morphology to that of the normal control group (Fig. 2e). No human cells in the organs of the injected group were detected using FISH analysis (Fig. 2f and Positive control in Supplementary Fig. 6a). In addition, serum biochemical examination and hematological parameters of the experimental animals, cynomolgus monkeys with intravenous injection and rabbits with intra-articular injection, remained within the normal range (Fig. 2g, Supplementary Table 4, 5). In summary, IMRCs administration in vivo has a good safety profile and no significant toxicity in rabbits and cynomolgus monkeys. IMRCs with intra-articular injection were retained in the knee joint cavity without migrating to other organs.

### Intra-articular injection of IMRCs repairs punch defects in the rabbits’ meniscus

The efficacy of this IMRCs-based therapy was further evaluated. At week 8 post-injection, samples of the entire meniscus of rabbits were collected for macroscopic observations and assessed using a semi-quantitative scale according to the previous report.^40^ The defective lesions in the control group were clearly visible with few regenerative tissues at week 8, while the lesions in the IMRCs group were filled with new cartilage tissue (Fig. 3c, left panel and Supplementary Fig. 5). Rudert’s macroscopic score in the IMRCs group was 6.44 ± 0.71, which was higher than that in the control group (3.89 ± 0.36, *P* = 0.014) (Fig. 3c, right panel). Hematoxylin-eosin (HE) staining of the normal meniscus tissue showed that the chondrocytes and collagen fibers were arranged neatly and clearly (Supplementary Fig. 6b). A larger volume of new HE-stained tissue was observed in the regenerative area in the IMRCs group, and most cells in this lacunar region showed round to oval-shaped chondrocyte morphology arranged in chains (Fig. 3d, green arrowhead).

Strongly positive Safranin-O staining (red) of extracellular matrix and glycosaminoglycan content was observed in the regenerated tissues in the IMRCs group at week 8, similar to the natural meniscus tissue (Supplementary Fig. 6b), whereas the control group showed comparatively weaker Safranin-O staining, indicating only a small amount of glycosaminoglycans (Fig. 3e).

Masson trichrome-stained sections were used to assess collagen distribution, orientation, and characterization of the matrix components. In a normal meniscus, a large number of collagen fibers were arranged in parallel. Meanwhile, chondrocytes, including some clusters of chondrocytes, were usually located in rows between fibrous bundles (Supplementary Fig. 6b). In the IMRCs group at week 8, collagen fibers were intertwined with normal fibers, with lots of round or oval-shaped fibro-chondrocytes similar to normal meniscus tissue. While chondrocytes in a homogeneous population were also observed (Fig. 3f, black arrowhead), suggesting that IMRCs promote fibrocartilage regeneration.

The histochemical staining of type I collagen (Col-1) and type II collagen (Col-2) fibers showed that Col-1 was lower in and around the repair foci, but Col-2 of repair foci was observed in the IMRCs group (Fig. 3g, h), which is similar to the expression distribution of Col-1 and Col-2 in the normal rabbit meniscus (Supplementary Fig. 6b). Statistical analysis showed that Col-2 in the repair foci was significantly higher in the IMRCs group (*P* = 0.036), while Col-1 expression was higher in the control group (*P* = 0.029) (Fig. 3i). We also performed negative control staining for Col-1, Col-2 (Supplementary Fig. 6c, d). These findings demonstrate that IMRCs promote collagen formation and help improve the composition of the regenerated meniscus tissue.

We further performed semi-quantitative histological scoring of the regenerated meniscus tissue using a modified Pauli scoring system,^41,42^ including regenerated tissue surface, tissue morphology, cellularity, collagen fiber organization, and matrix staining with Safranin-O (Detailed criteria in Supplementary Table 6). The IMRCs group scored 10.14 ± 1.34, significantly higher than that of the control group (4.29 ± 1.21, *P* = 0.010) (Fig. 3j). In summary, the above data suggest that the degree of meniscus regeneration in the IMRCs group is significantly higher than that in the control group.

### A phase I dose-escalating trial of IMRCs in the treatment of meniscus injury

#### Patient characteristics and treatment protocol

**Fig. 4:**
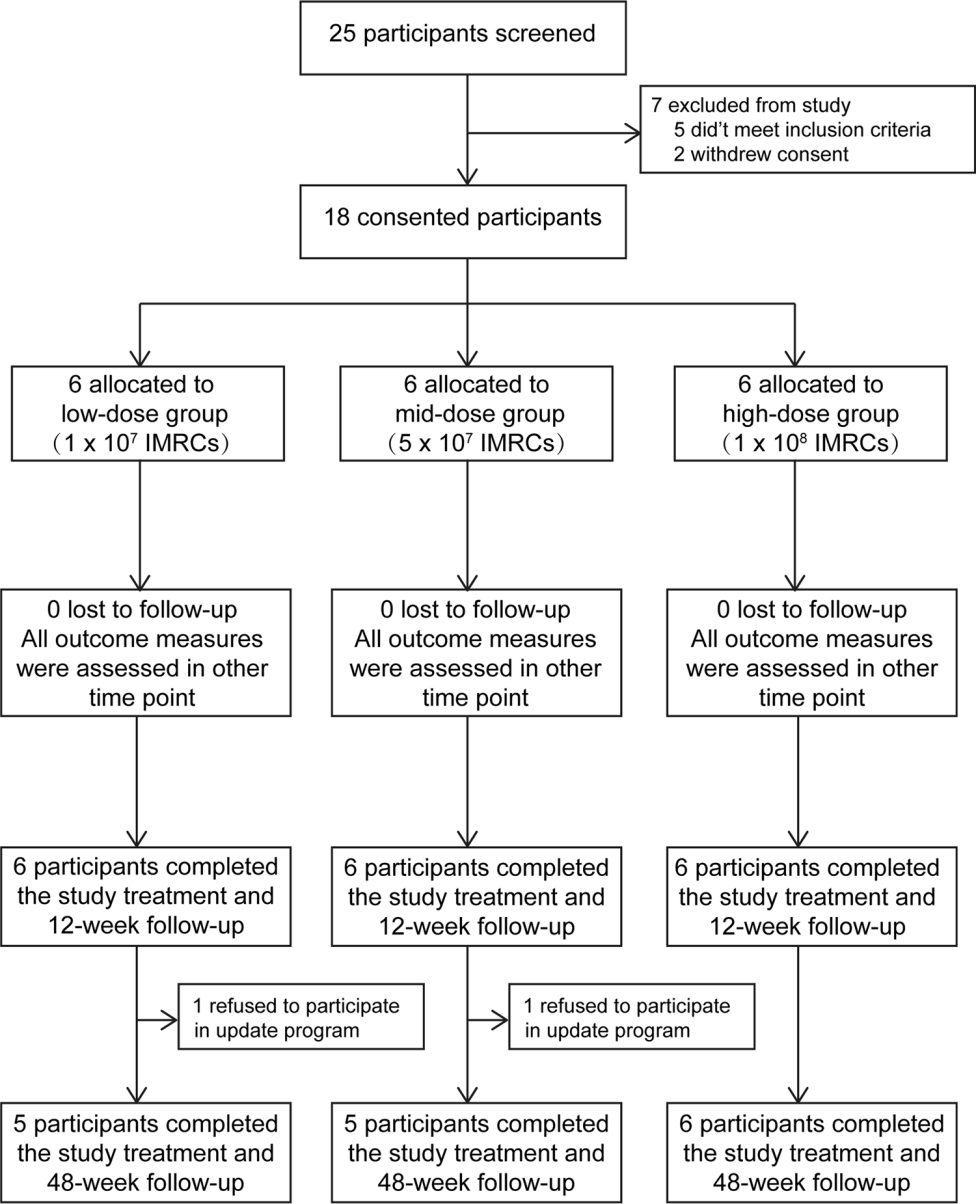
Study CONSORT diagram showing the process of subject participation

A phase I clinical trial of intra-articular injection of IMRCs in the treatment of meniscus injury was carried out at Tongji Hospital. From January 2019 to December 2020, twenty-five patients were screened for eligibility. Seven patients were excluded from the trial because five did not meet the inclusion criteria, and two declined to participate in this study. Chronologically, eighteen participants were allocated to 3 groups: 6 in the low-dose group (1 × 10^7^ cells/3 ml/knee), 6 in the mid-dose group (5 × 10^7^ cells/3 ml/knee), and 6 in the high-dose group (1 × 10^8^ cells/3 ml/knee). All 18 patients completed the 12-week of follow-up, and sixteen completed an updated 48-week follow-up after two participants dropped out due to personal reasons (Fig. 4). Patients in each group had similar demographic characteristics at baseline (Table 1).

**Table 1:** Demographics and baseline characteristics

Characteristics	Total Cohort (n = 18)	Low-dose group (n = 6)	Mid-dose group (n = 6)	High-dose group (n = 6)
Age (Median/range, years)	37 (27-64)	36 (27-64)	40 (32-62)	39 (27-55)
Gender				
Male	12	4	4	4
Female	6	2	2	2
Smoker (n)	2	2	0	0
Height (Mean ± SD, cm)	166.39 ± 8.81	166.33 ± 10.52	166.83 ± 5.85	166.00 ± 10.88
Weight (Mean ± SD, kg)	65.78 ± 13.52	68.92 ± 16.72	64.08 ± 10.62	64.33 ± 14.53
BMI (Mean ± SD, kg/m^2^)	23.64 ± 3.75	24.68 ± 1.14	22.91 ± 2.65	23.32 ± 4.65
Months since symptom onset (Median/range)	21 (6-120)	26 (6-72)	30 (6-120)	21 (12-96)
Knee laterality				
Left	8	5	2	1
Right	10	1	4	5
MRI of injected knee^a^				
I degree	2	0	1	1
II degree	16	6	5	5
Coexisting diseases (n)	4	0	3	1
Gout	2	0	2	0
Heart disease	1	0	0	1
Alimentary disease	1	0	1	0

*BMI* body-mass index, *MRI* magnetic resonance imaging

^a^According to the Stoller classification standard

#### Intra-articular injection of IMRCs is safe

All participants tolerated the injection procedure well, with no serious adverse events (SAE) associated with IMRCs during the post-procedure period. Four of the 18 patients (22.2%) experienced symptoms of mild adverse events (AEs), including joint pain after injection in 1 case of the mid-dose group and local swelling sensation in the knee joint in 3 cases of the high-dose group (Supplementary Table 7). However, the pain and swelling sensation was tolerable, and the MRI scans immediately following the complaints showed no abnormal signals. These mild symptoms disappeared without intervention, and no participants dropped out of the clinical trial because of AEs.

**Fig. 5:**
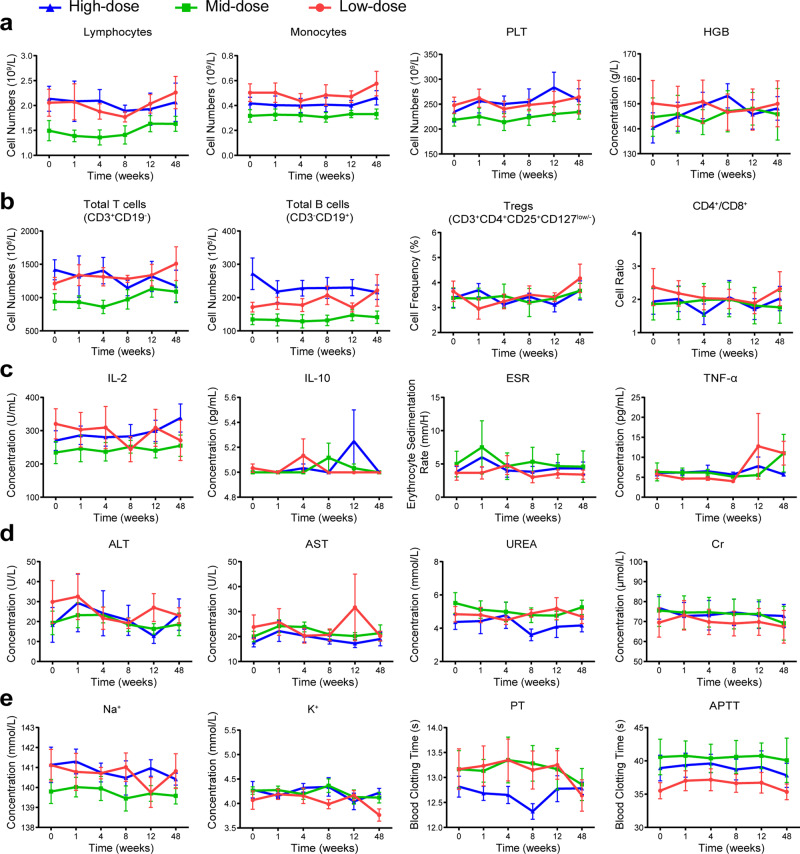
Clinical trial shows the safety profile of IMRCs by blood tests at six time points post-injection. **a** Conventional complete blood test. PLT, platelet. HGB, hemoglobin. **b** Functional analysis of human lymphocyte subsets. Treg: regulatory T lymphocyte. **c** Systematic inflammatory cytokines. IL, interleukin. TNG-α, tumor necrosis factor-alpha. ESR, erythrocyte sedimentation rate. **d**, **e** Basic metabolic panel, serum enzyme and blood clotting tests. ALT, alanine aminotransferase. AST, aspartate aminotransferase. Cr, creatine. PT, prothrombin time. APTT, activated partial thromboplastin time. For (**a)**–(**e**), n = 6 in each group. Data are presented as mean ± SEM

Blood tests, including complete blood count, basic metabolic panel, blood enzyme tests, blood clotting tests, human lymphocyte subsets and inflammatory cytokines, were almost in the normal reference range and showed no significant change after injection (Supplementary Table 8, 9). No signs of AEs such as anemia, thrombocytopenia, and activation of the mononuclear phagocyte system or allergic reactions were found due to the stable number of white blood cells, neutrophils, monocytes, platelets and hemoglobin (Fig. 5a). Human lymphocyte subsets containing the total T cells (CD3^+^), B cells (CD3^−^CD19^+^), NK cells (CD3^−^/CD16^+^CD56^+^), CD4^+^/CD8^+^, and regulatory T lymphocyte (CD3^+^CD4^+^CD25^+^CD127^low/−^) were not significantly altered (Fig. 5b), indicating the immune system was intact and unaffected by transplantation of IMRCs. In addition, there were no significant changes in critical inflammatory cytokines associated with systematic inflammatory responses, including interleukin (IL)-1-beta, IL-2, IL-6, tumor necrosis factor-alpha (TNF-α), erythrocyte sedimentation rate (Fig. 5c and Supplementary Table 8). Other clinical laboratory indicators, including alanine aminotransferase, aspartate aminotransferase, creatine, potassium, and international normalized ratio (INR), did not show significant changes (Fig. 5d, e), which indicated normal liver and kidney function, coagulation, and electrolyte balance after intra-articular IMRCs transplantation. Besides, there were no newly formed masses. No clinical deterioration or vital signs changes were reported during the study. In summary, intra-articular injection of IMRCs is a safe treatment strategy for patients with meniscus injuries.

#### Radiological analysis shows that IMRCs enhance meniscus repair

**Fig. 6:**
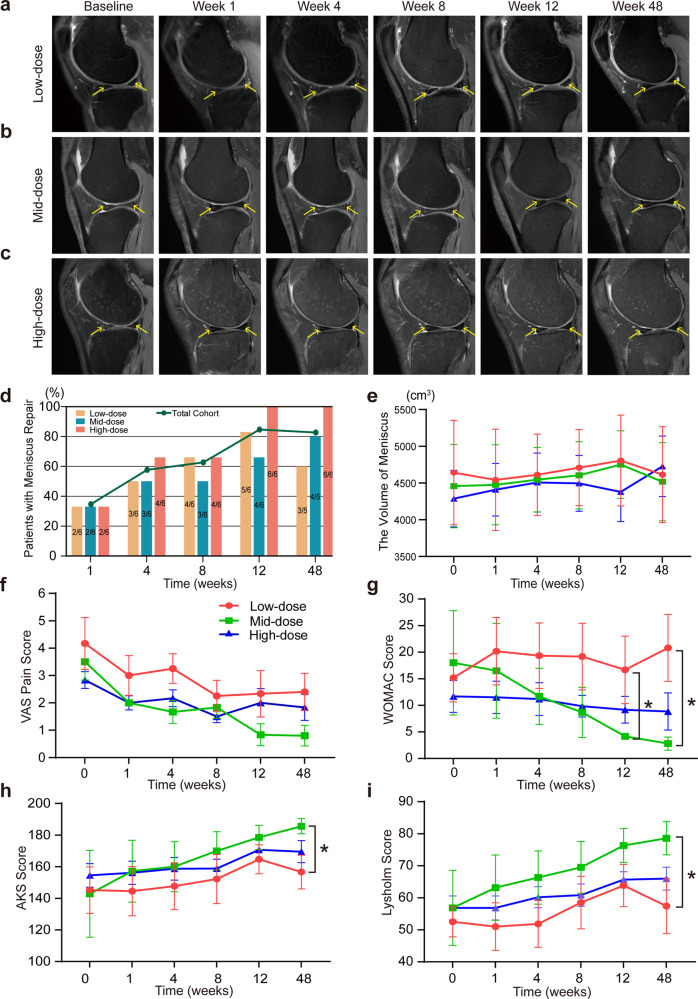
Clinical trial shows efficacy outcomes of IMRCs in MRI analysis and knee function scores after IMRCs injection. **a**–**c** Impairment of meniscus in knee MRI scans in different dose groups. **d** Percentage of patients with meniscus repair. **e** The volume analysis of the meniscus at six time points post-injection. **f**–**i** The change of knee function scores after IMRCs injection. VAS pain score (**f**), WOMAC score (**g**). AKS knee score (**h**), and Lysholm knee scale (**i**). VAS, Visual Analogue Score. WOMAC, Western Ontario and McMaster Universities Osteoarthritis Index. AKS, American Knee Society. For (**f**)–(**i**), n = 6 in each group. *P < 0.05; Data are presented as mean ± SEM

The efficacy of IMRCs was assessed using MRI according to the Stoller’s classification standard of meniscus injury.^43^ Before IMRCs transplantation, the injured meniscus showed line, strip, or plaque hyperintensity in the proton density (PD) -weighted imaging (WI) of sagittal MRI view. After IMRCs injection, the density of the meniscus was uniform, and the meniscal edges became regular, with reduced or disappeared hyperintensity of meniscus lesions on MRI view (Fig. 6a–c, triangle area indicated by yellow arrow), which means the meniscus has been repaired. Some patients even demonstrated almost complete disappearance of the torn meniscus hyperintensity (Patients 1, 7, 8, 9, 13 15, 17 in Supplementary Fig. 7–9).

Data from meniscus MRI images at week 1, 4, 8, 12 and 48 after IMRCs injection demonstrated that 6 (33.33%), 10 (55.56%), 11 (61.11%), 15 (83.33%) and 14 (81.25%) had healed meniscus. The meniscus repair rate at week 12 was significantly higher than that at week 1 (33.33% vs. 83.33%, *P* = 0.006). In the high-dose group, meniscus healing was observed in all 6 cases (6/6, 100%) at week 12, better than the low- and mid-dose groups. At week 48, the repair rate of meniscus was higher in the high-dose group (6/6, 100%) than in the low-dose group (3/5, 60%) and mid-dose group (4/5, 80%) (Fig. 6d). Generally, the high-dose group showed earlier regeneration and longer-lasting repair effect.

Meniscal volume was calculated by quantitative MRI evaluation using 3D Slicer software. Overall, meniscus volume improved after intra-articular injection of IMRCs in all three groups, which increased from 4288 mm^3^ to 4728 mm^3^ between baseline with week 48 in the high-dose group (*P* = 0.038) (Fig. 6e). Furthermore, meniscus volume peaked at week 12 in the low- and mid-dose groups and subsequently decreased, while meniscus volume continued to increase in the high-dose group over 48 weeks until the end of follow-up.

#### Intra-articular injection of IMRCs relieves pain and improves knee function

The pain intensity was assessed on a 10-point Visual Analogue Score (VAS). In a total of 18 cases, the VAS (described as mean ± SEM below) decreased from 3.50 ± 0.40 before treatment to 1.69 ± 0.33 at week 48 after IMRCs injection, and the VAS pain intensity in patients was significantly decreased compared to the baseline (week 1, *P* = 0.009; week 4, *P* = 0.010; week 8, *P* < 0.0001; week 12, *P* < 0.0001; week 48, *P* < 0.0001). These results suggested that IMRCs could alleviate knee pain in patients with meniscus injuries. In addition, The VAS pain score was lower in the mid-dose group than in the other two groups without significant difference (Fig. 6f).

The knee function was assessed by the Western Ontario and McMaster Universities Osteoarthritis Index (WOMAC) score, the Lysholm knee score, and the American Knee Society (AKS) knee score. The WOMAC score in 18 cases decreased from 14.94 ± 1.83 baseline to 10.81 ± 5.29 at week 48, a tendency that indicated improved outcome of knee function (Fig. 6g). Furthermore, the WOMAC score in the mid-dose group decreased significantly lower levels at week 12 (4.17 ± 0.60 vs. 16.67 ± 6.34, *P* = 0.041) and week 48 (2.80 ± 1.24 vs. 20.80 ± 6.28, *P* = 0.011), compared to the low-dose group. The AKS (Fig. 6h) and Lysholm knee scores (Fig. 6i) showed an upward tendency over time, suggesting enhanced knee function after intra-articular injection of IMRCs. Moreover, the mid-dose IMRCs group had a significant advantage over the low-dose group in terms of increased AKS score (185.60 ± 4.74 vs. 156.60 ± 10.68, *P* = 0.024) and higher Lysholm score (78.60 ± 5.17 vs. 57.40 ± 8.55, *P* = 0.024) at week 48. In summary, intra-articular injection of IMRCs is safe and relieves pain intensity and improves knee function. Moreover, the mid-dose IMRCs are more potent in improving the clinical function of the knee joint after meniscus injury.

## Discussion

The meniscus has limited healing capacity, especially the avascular zone that occupies two-thirds of the meniscus. We have shown that intra-articular injection of hESCs-derived IMRCs enables the endogenous regeneration of injured meniscus in rabbits. This is likely achieved by modulating the injured environment as the IMRCs possess strong immunomodulatory and pro-regenerative gene profiles in response to the synovial fluids from patients with meniscus injury. Our phase I clinical trial showed that intra-articular injection of IMRCs is not only safe but also beneficial based on improvement in symptoms and MRI imaging. Our dose-escalation study further identified an optimal dose for treatment, setting the foundation for further clinical studies.

Many pre-clinical studies suggested MSC-based regenerative treatment is a promising option to overcome poor intrinsic healing capacity, including synovial-derived MSCs (S-MSCs),^44–47^ bone marrow-derived MSCs (BM-MSCs),^48–50^ and adipose-derived MSCs (AD-MSCs).^51–53^ MSCs can promote meniscus healing in animal models either by differentiation into chondrocytes that resembled meniscus cartilage^48,51,53^ or via autocrine or paracrine pathways.^46,54^ In the present study, we found a remarkable repair of the injured meniscus, yet no human IMRCs were present in the meniscus of the rabbits, supporting the mechanism by which IMRCs promote cartilage regeneration is achieved via their secreted bioactive molecules. The absence of the IMRCs by 8 weeks post-injection also suggests the safety of the cells.

The substantial pro-regenerative capacity of the IMRCs prompted us to examine their properties and compare them with other types of MSCs, especially UCMSCs. In previous vitro studies, the levels of IL-6, MCP-1, PEG2, and TGF-β1 in UCMSCs supernatants were increased, and UCMSCs were able to significantly reduce the production of IL-6 and IL-12 when activated by M1 macrophages.^55^ Besides, UCMSCs could secrete more chemokines (e.g., RANTES, MIP-1β, MCP-1, IP-10), and inflammatory factors (e.g., IL-6, IL-8, IL-1RA).^56,57^ Different from UCMSCs, we found IMRCs secrete more anti-inflammatory factors (IL-1RA, LIF) and pro-regenerative factors (SDF-1α, IP-10, MIG, PDGF-BB) but fewer pro-inflammatory factors (GRO-α, IFN-γ, TNF-α, TNF-β, IL-8) when stimulated with the patient’s synovial fluid. Moreover, transcriptomics analysis also suggests the pro-regenerative potential of IMRCs as IMRCs highly express chondrocyte proliferation and vascular endothelial growth factors. Research has shown that SDF-1α plays a significant role in tissue regeneration by enhancing cell migration.^58^ In a study conducted on rats undergoing meniscus excision, intra-articular injection of SDF-1α resulted in an increased size of the reparative meniscus after six weeks, facilitated by the promotion of meniscus healing through macrophages.^59^ Additionally, the study demonstrated that SDF-1α promotes the expression of cell cycle protein D1 in chondrocytes, thereby facilitating chondrocyte proliferation via the Erk1/2 and NF-κB pathways.^60^ These properties of IMRCs explain why intra-articular injection of IMRCs results in a substantial repair of the injured meniscus.

With the safety profiles in rabbits and monkeys, we conducted the phase I clinical trial. Our results show that intra-articular injection of IMRCs is safe, similar to previous reports using various types of MSCs,^61^ However, the efficacy is difficult to compare mainly due to a wide range of variables in previous clinical studies, including the small sample size, various cell types, different cell dosages and intervention methods, short follow-up time, and outcome measures.^19–25^ Meanwhile, the stem cells used in previous clinical studies were derived from autologous tissue of different ages,^62^ leading to the prominent differences in these regeneration abilities, especially in aged persons. Therefore, it is necessary to do this single-center, open-label, dose-escalating clinical study for identifying the suitable cell type, dose, safety and efficacy. Besides using standard measures including VAS, WOMAC, AKS and Lysholm knee score together with MRI scans for effective analysis, we also tested the immune function containing human lymphocyte subsets and inflammatory cytokines in patients for safety profiles, and we found that the intra-articular injection of IMRCs provides therapeutic benefits to patients with meniscus injury without interference with the immune system.

Importantly, we identified an ideal dose (5 × 10^7^) of IMRCs for safe and effective treatment through a dose-escalation study. To our knowledge, this is the first dose-escalation study using three different cell dosages of MSCs/IMRCs treatment for meniscus injury. Previous clinical studies chose a dose ranging between 1 × 10^6^ and 1.5 × 10^8^ stem cells,^19,23^ resulted in different treatment outcomes. Our study clearly shows that although the high-dose group results in fast and better meniscus repair, it has more adverse effects (hence lower safety). The mid-dose (5 × 10^7^) has a better clinical improvement than low-dose group without obvious side effects. Our finding highlights a critical need for dose-escalation clinical study in IMRCs-mediated treatment for meniscus injury.

Taken together, despite the small sample size and absence of the control group, this study provides robust evidence that IMRCs are safe for meniscus injury and can promote meniscus regeneration and healing, as well as sufficient proof-of-concept (POC) data to justify further randomized, double-blind controlled clinical trials.

## Materials and methods

### Cell culture

The IMRCs were prepared as described previously, and they have been verified in accordance with the requirements of China’s National Institutes for Food and Drug Control (NIFDC).^26^ Briefly, IMRCs were derived from a clinical hESCs line (CB0019). IMRCs were passaged when they reached approximately 80% confluence in α-MEM medium (Gibco, 12561-049) supplemented with 5% KOSR, 1% Ultroser G (Pall corporation, New York, NY, USA; 15950-017), 1 × L-glutamine, 1 × NEAA, 5 ng/mL bFGF and 5 ng/mL TGF-β (Peprotech, 96-100-21-10). All cultures were maintained in a humidified incubator (Thermo Fisher Scientific, Waltham, MA, USA) at 37 °C, with 5% CO_2_ and atmospheric O_2_.

### Cell co-culture with synovial fluid stimulation

At the fourth passage, cells were digested and plated onto 12-well plates. IMRCs and UCMSCs were seeded at a density of 1 × 10^5^ cells per well. After 24 h of culture, the medium was changed to the “Stimulating Medium” composed of α-MEM supplemented with 5% KOSR, 1% Ultroser G, 1 × L-glutamine, 1 × NEAA, and 10% synovial fluid from meniscus-injured patients. Cells and supernatant were collected after synovial fluid stimulation for further analysis.

### Cytokine analysis

The supernatants of IMRCs and UCMSCs were collected after synovial fluid stimulation for 24 h. The samples were analyzed by 48-plex Bio-Plex Pro Human Cytokine Assay (Bio-Rad, Hercules, CA, USA; 1200728), following the guidelines provided by the manufacturer.

### Preparation and analysis of RNA-seq libraries

Total RNA was extracted from IMRCs and UCMSCs using Trizol (Invitrogen, Waltham, MA, USA; 15596018). Subsequently, RNA-seq libraries were prepared with the NEBNext^®^ Ultra^TM^ RNA Library Prep Kit for Illumina^®^ and subjected to paired-end sequencing with 150 bp reads on an Illumina HiSeq X-Ten sequencer. After filtering the sequencing data, we utilized STAR to map them to the hg38 reference genome. Gene expression levels were estimated by counting the reads mapped to genomic or exon regions, and FPKM (Fragments per Kilobase per Million Mapped Fragments) was used. DESeq2 was employed for differential gene expression analysis, with criteria set as |log2-fold change | ≥1 and *P* value < 0.05. Principal Component Analysis (PCA) was performed using the DESeq2 package in R. Gene Ontology analysis for DEGs was conducted using DAVID (version 6.8). Heatmap analysis was carried out using the heatmap.2 functions in R.

### Animals

All animal experiments were conducted according to protocols approved by the Animal Care and Use Committee of Tongji Hospital, Tongji Medical College of Huazhong University of Science and Technology (Registration number: TJH-201806001). 26 New Zealand white rabbits that were 6 months old and weighed 2.5-3.0 kg were obtained from Wanqian Jiaxing Biotechnology Co., Ltd., Hunan, China. After arrival, all rabbits were acclimated to the animal facility at least 7 days before the experiments were initiated. The rabbits were housed in single cages and kept in a 12-hour light/12-hour dark cycle at 22 ± 2 °C with water and food ad libitum.

### Animal surgery and post-surgery care

All rabbits fasted for 12 h before surgery. In sterile settings, a knee arthrotomy was performed under general anesthesia (RDW Life Science Co., Ltd., isoflurane, R510-22-10). Anesthesia was supplied with a small oxygen mask through a veterinary isoflurane tank (RDW Life Science Co., Ltd., R580). The anesthesia was induced at 4-5% with full airflow and maintained at 3-3.5%. The surgical area was shaved and rinsed with an antiseptic fluid. A sterile skin biopsy punch machine (Integra Life Sciences Production Corporation, 33-31 A) was used to create a 1.5 mm full-thickness, cylindrical, vertical defect at the anteromedial part of the right leg medial meniscus. The operated knee was bandaged with a loose non-elastic bandage and left mobile in their cages for 24 h after surgery. Antibiotics and antiviral medicine were given immediately after surgery and continued for three consecutive days post-surgery (benzylpenicillin Sodium, North China Pharmaceutical Group Co., Ltd.; Aciclovir, Hubei Wushi Pharmaceutical Co., Ltd.). The antibiotics and antivirals were dissolved in normal saline; the dosages were as follows: benzylpenicillin Sodium 200,000 units/day, Aciclovir 2 ml/day (one vial dissolved in 5 ml normal saline).

### Cell transplantation and immunosuppression in rabbits

To evaluate their safety over both short and long durations, we conducted a series of biosafety experiments following the ‘Guidelines for Human Somatic Cell Therapies and Quality Control of Cell-based Products’ issued by the China Food and Drug Administration (CFDA).

The IMRCs stored in a liquid nitrogen tank were thawed in a cell thawing system (Biocision, BCS-602) for 2-3 min at 37 °C. After thawing, a single dose of 1.0 × 10^7^ IMRCs was injected using a 27-gauge sterile needle into the operated knee (right knee). The injected knee was kept straight for a few minutes, and the rabbits were left free in their cages under observation for 24 h. In the control group, a similar amount of normal saline was administered to the operated knees. All groups received daily immunosuppression with Tacrolimus (MedChemExpress, HY-13756A), which began three days before injecting IMRCs. The FK506 was given subcutaneously every day at a dose of 0.05 mg/kg/day until the time point in all groups.

### Tissue harvesting and processing in rabbits

The rabbits were sacrificed by overdosage with isoflurane 8 weeks after IMRCs injection with anesthesia. After sacrificing, the organs and whole meniscus were placed in 4% ice-cold PFA. Images of the whole meniscus were taken on the same day of harvesting and further completed the dehydration, paraffin embedding, and sectioning of the tissues.

### Macroscopic observation and semiquantitative scoring

Tissue growth was assessed macroscopically using naked-eye observation and entire meniscus images were obtained under a microscope (Guangzhou Micro-shot Technology Co., Ltd., MZ62). The meniscus defect filling and quality of repair were assessed. The defect repair and other features of repair were scored with a semiquantitative scale adopted from Rudert et al,^40^ originally used for cartilage healing, the minimum score is 3, and the maximum is 8.

### Histological evaluation and immunohistochemistry staining of the regenerated tissue

All the tissues were sliced with microtome at 4 μm thickness. To detect meniscus cells (chondrocytes) in the neo-meniscus and analyze collagen distribution, matrix stainability and matrix contents, glycosaminoglycan, H&E staining, Masson’s trichrome and Safranin-O staining were used. For toxicity analysis, H&E staining was used to examine the morphology of key organs. Immunostaining for type I & II collagen in the regenerated meniscus was carried out to show the expression and distribution of collagen. Sections were incubated with the following primary antibodies: Collagen I Antibody (Col-1) (1:100; GeneTex, GTX26308), Anti-Collagen Type II (Ab-1) mouse mAb (II-4C11) (1:200; Sigma-Aldrich, CP18-100UG). Immunostainings were finished using Goat anti-mouse secondary antibody (1:1000; Servicebio, G1214).

For quantification of histology for regenerated meniscus, Pauli’s scoring system was used as the previous report,^41,42^ and this scale assesses different aspects of the tissue histology including regenerated tissue surface, cellularity, collagen fiber organization, and matrix stainability with safranin-O. After staining, all sections were photographed under a microscope (Hamamastu, NanoZoomer S360).

### Fluorescence in situ hybridization (FISH) analysis for existing of IMRCs

Human DNA-specific reference probes linked to fluorescent molecules, i.e., FISH analysis was used to detect human cells in the rabbit meniscus and internal organs according to previous reports.^63^ Briefly described as following steps: Firstly, tissues were pre-processed by baking, dewaxing, washing, permeabilization, enzymatic digestion, dehydration and drying. Then drop 10 μl the probe (Wuhan HealthCare Biotechnology Co., Ltd., CEP Y/CEP X dual-color probe) on the hybridization area; after sealing the cover glass, the glass slides were placed on the hybridization instrument (Hangzhou Rui Cheng Instrument Co., Ltd., SH2000), co-denatured at 85 °C for 5 min (hybridization instrument should be preheated to 85 °C), and hybridized at 42 °C for 2-16 h. Finally, the glass slides were washed and counterstained in a dark room. Positive staining which means human cells were observed under a laser confocal microscope (Olympus, FV3000).

### Dose-escalating clinical trial design and ethical considerations

The study is an open-label, dose-escalation phase I clinical trial conducted from January 2019 to December 2020 that investigated the role of human embryonic stem cells-derived mesenchymal stem cells (IMRCs) in the treatment of meniscus injury (ClinicalTrials.gov Identifier: NCT03839238). The protocol was approved by the ethics committee of the Tongji Hospital, Tongji Medical College, Huazhong University of Science and Technology, Wuhan, China (certificate of approval number: TJ-IRB20180901). According to the suggestion of the National Health Commission, we extended the follow-up time from 12-week to 48-week and obtained updated approval from the ethics committee (updated certificate of approval number: TJ-IRB20190911). This study was conducted according to the Declaration of Helsinki and Good Clinical Practice principles. All patients provided written informed consent.

### Participants Eligibility

25 patients were screened. Eligible trial subjects were adults (18-65 years old) with Grade I-II meniscus injury according to the Stoller classification standard^43^ in MRI. At least two professional radiologists confirmed the meniscus injury. Furthermore, the patients had pain or knee function impairment after accepting three months of nonoperative treatment. The investigators reviewed the age, symptoms, medical history, MRI, and inclusion and exclusion criteria. If the investigator judged that the poor physical conditions which were unfavorable for the intra-articular injection and follow-up, or if there were evidence of severe meniscus injury that needed surgical operation, the patients would be excluded. The details of the screening criteria are provided in the Supplementary Materials and Methods.

### Allocation and Interventions

Eighteen patients enrolled in our study were sequence assigned to three groups: 6 patients in each of the three-group received 1 **×** 10^7^/3 ml, 5 **×** 10^7^/3 ml, and 1 **×** 10^8^/3 ml IMRCs intra-articular injection. The IMRCs were suspended in an electrolyte solution. The processes of intra-articular injection operation were performed in a dedicated room and meeting the aseptic principles. Clinical follow-up was carried out at week 1, week 4, week 8, week 12, and week 48 after injection. Physical examination, adverse events, blood and urine tests, ultrasound, MRI, and clinical outcome measures were recorded at each time point.

### Outcome measures

#### Primary outcome

Safety was measured by the documentation of local and systemic adverse events. A combination of vital signs, physical examination, and blood test (including the routine manual complete blood count, biochemistry, liver, kidney function, electrolytes, coagulation, cellular immunity, lymphocyte function and cytokines) and urine laboratory tests were used at 1, 4, 8, 12, and 48 weeks. Adverse events were categorized using the National Cancer Institute Common Terminology Criteria for Adverse Events version 4.0 scale (NCI-CTCAE 4.0).

#### Secondary outcomes

Secondary outcomes included patient-reported clinical outcomes and radiological assessments.

1. **Clinical outcomes**. Variation of pain intensity was assessed using the 10-point VAS pain score. The changes in knee functions and disease-specific quality of life were evaluated by the Western Ontario and McMaster Universities Osteoarthritis Index (WOMAC) score, Lysholm knee scale score, and the American Knee Society (AKS) knee score.^64^ The WOMAC score is one of the most commonly used worldwide for patient-reported outcome measurement in patients with lower limb osteoarthritis.^65,66^ The AKS knee score is another reliable index for evaluating knee function including knee joint pain, mobility and stability.^67^ And the Lysholm knee scale is a condition-specific outcome measure originally designed to assess chondral disorders of the knee.^68^
2. **Radiological outcomes**. The MR images of the meniscus were evaluated by two professional radiologists according to the Stoller classification standard.^43^ The injured meniscus were classified into three grades, grade I corresponds to punctate elevated signals with no connection to the meniscal surface, while grade II indicates a linear signal elevation with no contact with the articular meniscal surface, and grade III indicates a linear signal elevation with at least one point of contact with the meniscal surface.^69^

### Statistical analysis

SPSS 23.0 software was used for the statistical analysis. Outcome measures were analyzed based on the intention-to-treat population. Data are reported as means ± SEM. An unpaired t-test was used to assess efficacy before and after injection, and a one-way analysis of variances was used for the comparison in three groups. Two-tailed Mann-Whitney tests were used for those with unequal sample sizes and outcome measures that were not normally distributed. To identify clinical function changes between baseline and 1, 4, 8, 12, and 48 weeks follow-up for self-reported knee scores, the two-way ANOVA was performed. Within-group differences were analyzed per the Mann-Whitney U and Wilcoxon signed-rank tests, respectively. Statistical significance was determined at *P* < 0.05. Statistical analyses were performed using GraphPad Prism 8 software.

### Supplementary information

Supplementary Materials

Supplementary Table 2

## Data Availability

The data used in the current study are available from the corresponding authors upon reasonable request. Participants data without names and identifiers will be made available after approval from all corresponding authors. Please refer to Supplementary Materials and Methods for further details regarding the materials and methods used.

## References

[1] Logerstedt DS, Snyder-Mackler L, Ritter RC, Axe MJ (2010). Knee pain and mobility impairments: meniscal and articular cartilage lesions. J Orthop Sports Phys. Ther..

[2] Arnoczky SP, Warren RF (1982). Microvasculature of the human meniscus. Am J Sports Med.

[3] Fox AJ, Bedi A, Rodeo SA (2012). The basic science of human knee menisci: structure, composition, and function. Sports Health..

[4] Makris EA, Hadidi P, Athanasiou KA (2011). The knee meniscus: structure-function, pathophysiology, current repair techniques, and prospects for regeneration. Biomaterials..

[5] Rodkey WG (2000). Basic biology of the meniscus and response to injury. Instr Course Lect.

[6] Scotti C, Hirschmann MT, Antinolfi P, Martin I, Peretti GM (2013). Meniscus repair and regeneration: review on current methods and research potential. Eur Cell Mater.

[7] Sihvonen R (2013). Arthroscopic partial meniscectomy versus sham surgery for a degenerative meniscal tear. N Engl J Med..

[8] Andersson-Molina H, Karlsson H, Rockborn P (2002). Arthroscopic partial and total meniscectomy: A long-term follow-up study with matched controls. Arthroscopy..

[9] Seil R, Becker R (2016). Time for a paradigm change in meniscal repair: save the meniscus!. Knee Surg Sports Traumatol Arthrosc.

[10] Rhim HC (2021). Mesenchymal stem cells for enhancing biological healing after meniscal injuries. World J Stem Cells..

[11] Cui X, Hasegawa A, Lotz M, D’Lima D (2012). Structured three-dimensional co-culture of mesenchymal stem cells with meniscus cells promotes meniscal phenotype without hypertrophy. Biotechnol Bioeng..

[12] Wang M, Yuan Q, Xie L (2018). Mesenchymal Stem Cell-Based Immunomodulation: Properties and Clinical Application. Stem Cells Int..

[13] Centeno CJ (2008). Regeneration of meniscus cartilage in a knee treated with percutaneously implanted autologous mesenchymal stem cells. Med Hypotheses..

[14] L PK (2019). The mesenchymal stem cell secretome: A new paradigm towards cell-free therapeutic mode in regenerative medicine. Cytokine Growth Factor Rev.

[15] Noel D, Djouad F, Jorgense C (2002). Regenerative medicine through mesenchymal stem cells for bone and cartilage repair. Curr Opin Investig Drugs.

[16] Tarafder S (2018). Engineered Healing of Avascular Meniscus Tears by Stem Cell Recruitment. Sci Rep..

[17] Lee WY, Wang B (2017). Cartilage repair by mesenchymal stem cells: Clinical trial update and perspectives. J Orthop Translat.

[18] Oryan A, Kamali A, Moshiri A, Baghaban EM (2017). Role of Mesenchymal Stem Cells in Bone Regenerative Medicine: What Is the Evidence?. Cells Tissues Organs..

[19] Vangsness CJ (2014). Adult human mesenchymal stem cells delivered via intra-articular injection to the knee following partial medial meniscectomy: a randomized, double-blind, controlled study. J Bone Joint Surg Am..

[20] Khalifeh SS (2019). Safety and efficacy of allogenic placental mesenchymal stem cells for treating knee osteoarthritis: a pilot study. Cytotherapy..

[21] Pak J, Lee JH, Lee SH (2014). Regenerative repair of damaged meniscus with autologous adipose tissue-derived stem cells. Biomed Res Int..

[22] Centeno CJ (2008). Increased knee cartilage volume in degenerative joint disease using percutaneously implanted, autologous mesenchymal stem cells. Pain Physician..

[23] Whitehouse MR (2017). Repair of Torn Avascular Meniscal Cartilage Using Undifferentiated Autologous Mesenchymal Stem Cells: From In Vitro Optimization to a First-in-Human Study. Stem Cells Transl. Med..

[24] Onoi Y (2019). Second-look arthroscopic findings of cartilage and meniscus repair after injection of adipose-derived regenerative cells in knee osteoarthrits: Report of two cases. Regen Ther..

[25] Sekiya I (2019). Additional Use of Synovial Mesenchymal Stem Cell Transplantation Following Surgical Repair of a Complex Degenerative Tear of the Medial Meniscus of the Knee: A Case Report. Cell Transplant..

[26] Wu J (2020). Immunity-and-matrix-regulatory cells derived from human embryonic stem cells safely and effectively treat mouse lung injury and fibrosis. Cell Res..

[27] Gu Q (2017). Accreditation of Biosafe Clinical-Grade Human Embryonic Stem Cells According to Chinese Regulations. Stem Cell Reports..

[28] Wu J (2020). First case of COVID-19 infused with hESC derived immunity- and matrix-regulatory cells. Cell Prolif.

[29] Liu J (2021). Infusion of hESC derived Immunity-and-matrix regulatory cells improves cognitive ability in early-stage AD mice. Cell Prolif.

[30] Zhao Y (2022). Human ESC-derived immunity- and matrix- regulatory cells ameliorated white matter damage and vascular cognitive impairment in rats subjected to chronic cerebral hypoperfusion. Cell Prolif.

[31] Xing D (2021). Clinical-Grade Human Embryonic Stem Cell-Derived Mesenchymal Stromal Cells Ameliorate the Progression of Osteoarthritis in a Rat Model. Molecules..

[32] Yang S (2022). Every road leads to Rome: therapeutic effect and mechanism of the extracellular vesicles of human embryonic stem cell-derived immune and matrix regulatory cells administered to mouse models of pulmonary fibrosis through different routes. Stem Cell Res. Ther..

[33] Hu W (2023). Secretome of hESC-Derived MSC-like Immune and Matrix Regulatory Cells Mitigate Pulmonary Fibrosis through Antioxidant and Anti-Inflammatory Effects. Biomedicines..

[34] Zhang X, Wu S, Zhu Y, Chu CQ (2021). Exploiting Joint-Resident Stem Cells by Exogenous SOX9 for Cartilage Regeneration for Therapy of Osteoarthritis. Front Med (Lausanne).

[35] Haseeb, A. et al. SOX9 keeps growth plates and articular cartilage healthy by inhibiting chondrocyte dedifferentiation/osteoblastic redifferentiation. *Proc Natl Acad Sci USA*. **118** (2021). 10.1073/pnas.2019152118 PMC7923381 33597301

[36] van Gastel N (2020). Lipid availability determines fate of skeletal progenitor cells via SOX9. Nature..

[37] Chen Y (2019). Sustained Release SDF-1alpha/TGF-beta1-Loaded Silk Fibroin-Porous Gelatin Scaffold Promotes Cartilage Repair. ACS Appl Mater Interfaces.

[38] Zhang W (2013). The use of type 1 collagen scaffold containing stromal cell-derived factor-1 to create a matrix environment conducive to partial-thickness cartilage defects repair. Biomaterials..

[39] Cui Z (2022). Endothelial PDGF-BB/PDGFR-beta signaling promotes osteoarthritis by enhancing angiogenesis-dependent abnormal subchondral bone formation. Bone Res..

[40] Rudert M, Wilms U, Hoberg M, Wirth CJ (2005). Cell-based treatment of osteochondral defects in the rabbit knee with natural and synthetic matrices: cellular seeding determines the outcome. Arch Orthop Trauma Surg..

[41] Pauli C (2011). Macroscopic and histopathologic analysis of human knee menisci in aging and osteoarthritis. Osteoarthritis Cartilage.

[42] Hatsushika D (2013). Intraarticular injection of synovial stem cells promotes meniscal regeneration in a rabbit massive meniscal defect model. J Orthop Res..

[43] Stoller DW, Martin C, Crues JR, Kaplan L, Mink JH (1987). Meniscal tears: pathologic correlation with MR imaging. Radiology..

[44] Horie M (2012). Implantation of allogenic synovial stem cells promotes meniscal regeneration in a rabbit meniscal defect model. J Bone Joint Surg Am..

[45] Ozeki N (2021). Synovial mesenchymal stem cells promote the meniscus repair in a novel pig meniscus injury model. J Orthop Res..

[46] Kondo S (2017). Transplantation of autologous synovial mesenchymal stem cells promotes meniscus regeneration in aged primates. J Orthop Res..

[47] Katagiri H (2013). Transplantation of aggregates of synovial mesenchymal stem cells regenerates meniscus more effectively in a rat massive meniscal defect. Biochem Biophys Res Commun.

[48] Zellner J (2017). Autologous mesenchymal stem cells or meniscal cells: what is the best cell source for regenerative meniscus treatment in an early osteoarthritis situation?. Stem Cell Res. Ther..

[49] Yuan X (2017). Stem cell delivery in tissue-specific hydrogel enabled meniscal repair in an orthotopic rat model. Biomaterials..

[50] Caminal M (2014). Use of a chronic model of articular cartilage and meniscal injury for the assessment of long-term effects after autologous mesenchymal stromal cell treatment in sheep. N Biotechnol.

[51] Ruiz-Iban MA (2011). The effect of the addition of adipose-derived mesenchymal stem cells to a meniscal repair in the avascular zone: an experimental study in rabbits. Arthroscopy..

[52] Moradi L (2017). Regeneration of meniscus tissue using adipose mesenchymal stem cells-chondrocytes co-culture on a hybrid scaffold: In vivo study. Biomaterials..

[53] Qi Y (2016). Targeted transplantation of iron oxide-labeled, adipose-derived mesenchymal stem cells in promoting meniscus regeneration following a rabbit massive meniscal defect. Exp Ther Med..

[54] Ando Y (2014). Stem cell-conditioned medium accelerates distraction osteogenesis through multiple regenerative mechanisms. Bone..

[55] Islam A, Urbarova I, Bruun JA, Martinez-Zubiaurre I (2019). Large-scale secretome analyses unveil the superior immunosuppressive phenotype of umbilical cord stromal cells as compared to other adult mesenchymal stromal cells. Eur Cell Mater.

[56] Amable PR, Teixeira MV, Carias RB, Granjeiro JM, Borojevic R (2014). Protein synthesis and secretion in human mesenchymal cells derived from bone marrow, adipose tissue and Wharton’s jelly. Stem Cell Res. Ther..

[57] Dabrowski FA (2017). Comparison of the paracrine activity of mesenchymal stem cells derived from human umbilical cord, amniotic membrane and adipose tissue. J Obstet Gynaecol Res..

[58] Hattori K (2001). Plasma elevation of stromal cell-derived factor-1 induces mobilization of mature and immature hematopoietic progenitor and stem cells. Blood..

[59] Nishida Y (2020). Intra-Articular Injection of Stromal Cell-Derived Factor 1alpha Promotes Meniscal Healing via Macrophage and Mesenchymal Stem Cell Accumulation in a Rat Meniscal Defect Model. Int J Mol. Sci..

[60] Kim GW (2015). CXC chemokine ligand 12a enhances chondrocyte proliferation and maturation during endochondral bone formation. Osteoarthritis Cartilage.

[61] Zhou YF, Zhang D, Yan WT, Lian K, Zhang ZZ (2022). Meniscus Regeneration With Multipotent Stromal Cell Therapies. Front Bioeng Biotechnol.

[62] Pak J, Chang JJ, Lee JH, Lee SH (2013). Safety reporting on implantation of autologous adipose tissue-derived stem cells with platelet-rich plasma into human articular joints. BMC Musculoskelet Disord.

[63] Svensson B (2011). Injection of human mesenchymal stem cells improves healing of vocal folds after scar excision–a xenograft analysis. Laryngoscope..

[64] Wright RW (2009). Knee injury outcomes measures. J Am Acad Orthop Surg.

[65] Bellamy N, Buchanan WW, Goldsmith CH, Campbell J, Stitt LW (1988). Validation study of WOMAC: a health status instrument for measuring clinically important patient relevant outcomes to antirheumatic drug therapy in patients with osteoarthritis of the hip or knee. J Rheumatol..

[66] Walker LC, Clement ND, Deehan DJ (2019). Predicting the Outcome of Total Knee Arthroplasty Using the WOMAC Score: A Review of the Literature. J Knee Surg..

[67] Liow RY, Walker K, Wajid MA, Bedi G, Lennox CM (2000). The reliability of the American Knee Society Score. Acta Orthop Scand.

[68] Kocher MS, Steadman JR, Briggs KK, Sterett WI, Hawkins RJ (2004). Reliability, validity, and responsiveness of the Lysholm knee scale for various chondral disorders of the knee. J Bone Joint Surg Am..

[69] Bick F (2019). The medial open-wegde osteotomy generates progressive intrameniscal integrity changes in the lateral knee compartment: a prospective MR-assessment after valgic osteotomy in the varus gonarthritic knee. Knee Surg Sports Traumatol Arthrosc..

