# Supplementary material for: Immunity-and-matrix-regulatory cells enhance cartilage regeneration for meniscus injuries: a phase I dose-escalation trial
Source: Signal Transduct Target Ther. 2023 Nov 1;8:417. doi: 10.1038/s41392-023-01670-7 (PMC10618459; doi:10.1038/s41392-023-01670-7)
Supplement: Supplementary file 1 — Supplementary Materials [file 41392_2023_1670_MOESM1_ESM.docx]

Supplementary Materials for

Immunity-and-matrix-regulatory cells enhance cartilage regeneration for meniscus injuries: a phase Ⅰ dose-escalation trial

Liangjiang Huang^1#^, Song Zhang^1#^, Jun Wu^2, 6#^, Baojie Guo^2#^, Tingting Gao^2^, Sayed Zulfiqar Ali Shah^1^, Bo Huang^3^, Yajie Li^1,4^, Bo Zhu^5^, Jiaqi Fan^6^, Liu Wang^2, 6, 7^, Yani Xiao^8^, Wenjing Liu^2^, Yao Tian^2^, Zhengyu Fang^1^, Yingying Lv^1^, Lingfeng Xie^1^, Sheng Yao^1^, Gaotan Ke^3^, Xiaolin Huang^1^, Ying Huang^8^, Yujuan Li^9^, Yi Jia^9^, Zhongwen Li^2, 6^, Guihai Feng^2, 6, 7^, Yan Huo^8^, Wei Li^2, 6, 7^, Qi Zhou^2, 6, 7^, Jie Hao^2, 6, 7^*, Baoyang Hu^2, 6, 7^*, Hong Chen^1,4^*

Correspondence to: hong chen ([chenhong1129@hust.edu.cn](mailto:chenhong1129@hust.edu.cn)); Jie Hao ([haojie@ioz.ac.cn);](mailto:haojie@ioz.ac.cn,) Baoyang Hu ([byhu@ioz.ac.cn](mailto:byhu@ioz.ac.cn)).

**This PDF file includes:**

Materials and Methods

Figures. S1-7

Tables S1 and S3-10

**Other Supplementary Materials for this manuscript include the following:**

Table S2

Materials and Methods

**Isolation and culture of UCMSCs**

UCMSCs, sourced from full-term newborn umbilical cords at the National Stem Cell Resource Center, Beijing, China, were cultured in α-MEM supplemented with 5% KOSR, 1% Ultroser G, 1 × L-glutamine, 1 × NEAA and 5 ng/mL bFGF. All cultures were incubated in a humidified incubator at 37 ℃, 5% CO_2_ and atmospheric O_2_. UCMSCs were passaged upon reaching approximately 80% confluence.

**The process of synovial fluid collection**

An independent in vitro experiment was designed to validate the response of IMRCs cells when stimulated by patients’ synovial fluid, which was approved by the ethics committee of Tongji Hospital (Approval No: TJ-IRB20220405). We selected four donors who underwent arthroscopy or surgery for meniscus injury in Tongji Hospital between May 1, 2022, and June 30, 2022. We collected the synovial fluid during the surgical process, which would have been discarded regularly. The synovial fluid was immediately centrifuged at 3000 rpm for 10 minutes. After centrifugation, the precipitate was removed, and the supernatant was retained as pure synovial fluid without red blood cells or other impurities. Then, the synovial fluid was frozen and stored at -80 °C for further co-culturing with IMRCs and UCMSCs.

**IMRCs inhibition of PBMC proliferation**

Human peripheral blood mononuclear cells (PBMCs) were thawed, resuspended in RPMI 1640 medium with 10% FBS and 1% penicillin-streptomycin, and seeded at 1 × 10^5^ cells per well in 96-well plates. PBMC activation was achieved using CD3/CD28 beads and IL-2. IMRCs were added at varying densities to the activated PBMCs. A control group with PBMCs at the same density but without IMRCs served as a reference (n = 3). On the fourth day of culture, we assessed PBMC proliferation using the CCK-8 assay.

**Chondrocytes [separation](javascript:;)**

Neonatal SD rats (3-5 days) were sacrificed and soaked in 75% alcohol for 15 minutes. The lower limbs of the rats were clipped and transferred to a 10 cm petri dish containing PBS solution. The articular meniscus cartilage tissue was separated, then cut into pieces and transferred to a 10 ml sterile centrifuge tube, centrifuged at 1200 rpm for 7 minutes. The upper PBS solution was discarded, and 1 ml 0.25% pancreatic enzyme was added , digested at 37 ℃ for 30 minutes. Recentrifuged at 1200 rpm for 5 minutes, removed the supernatant and added 3 ml type II collagenase solution, thoroughly blow and mixed the precipitate, then continued to add 3 ml of type II collagenase solution, thoroughly mixed and placed in the incubator at 37 ℃ for 6 hours digestion. Gently blew the adherent cells and transferred them into a 10 ml centrifuge tube, centrifuged at 1200 rpm for 7 minutes. Finally, removed the supernatant and the precipitated cells were meniscus chondrocytes.

**Chondrocytes co-culture with IMRCs conditioned medium**

Chondrocytes were thawed and seeded onto 6-well plates at a density of 1 × 10^5^ cells/well. On the next day, chondrocytes are stimulated by mixing them in a 1:1 ratio of chondrocyte culture medium and IMRCs conditioned medium for 24 hours. Than collected the cells for RNA-seq or qPCR analysis. The *SOX9* primer was used for the specific amplification: *SOX9* forward primer: 5'-CATCAAGACGGAGCAACTGA-3, and reverse primer: 5'-TCTGGTGGTCGGTGTAGTCA-3.

**Chondrocytes migration**

Primary rat chondrocytes were suspended in a culture medium at 200,000 cells/ml and 70 μl cell suspensions were pipetted into each chamber of the cell culture insert (ibidi, Munich, Germany; 81176) and incubated at 37 ℃ and 5% CO_2_ for 24 hours. The cell density was observed under a microscope, and the Culture-Insert 2 well was carefully removed using sterile forceps. The fresh culture medium was then replaced, and the cells were further incubated for 20 hours. Subsequently, images were captured using a microscope, and analysis was performed using ImageJ software.

**IMRCs tracking by MicroPET/CT**

IMRCs were labeled with 89Zr-oxine following a previously reported method. Whole-body imaging was performed using a micro-PET/CT (SINO UNION, Suzhou, China). Six New Zealand rabbits (three male and three female) were anesthetized with isoflurane. After a single right knee intra-articular injection of 1.5 × 10^7^ 89Zr-oxine-IMRCs (153.7 ± 19.2 μCi/rabbit), static whole-body micro-PET/CT scans were performed at 2, 24, 72, 168, 240, 336, and 504 hours after intra-articular injection (one rabbit died of anesthesia accident at 336h). After reconstructing the scanned raw data, the image and data analyses were carried out using the PMOD software by a statistical investigator blinded to the experimental design and grouping. The brain, heart, lung, liver, kidney, spleen, left knee joint, right knee joint (administration site), tibia, spine, muscle, intestine and bladder were delineated as regions of interest (ROIs). The ROIs were delineated by manual selection of the statistical investigator blinded to the experimental design in the reconstructed spatial images. After delineation, the radioactivity values of the ROIs per unit volume were obtained, and the SUV of each organ was calculated according to the radioactivity value of ROIs, weight and administration radioactive dose. $\text{SUV=}\frac{\text{The concentration of radioactivity in ROIs (μCi/g)}}{\text{Administration radioactive dose }\left（ \text{μCi} \right）\text{/weight (g)}}$. In order to scan and quantify the amount of radiation in various organs of the whole body at different time points, 15 minutes were required to scan the whole-body layer by layer at each time point, static whole-body layer-by-layer scanning and acquisition was used in this study.

**Monkeys**

Adult female and male cynomolgus monkeys (3-5 years old) were housed in single quarters with a 12/12 hours light/dark cycle. Animals were provided with monkey chow, enrichment food twice a day and water ad libitum. The monkey experiments were performed at the National Center for Safety Evaluation of Drugs, National Institutes of Food and Drug Control (Beijing, China). The Institutional Animal Care and Use Committee of the National Center for Safety Evaluation of Drugs approved all monkey studies (NO. IACUC-2018-k001). All animals were randomized using a random number table. The assessors were blind to all groups, and all outcome analyses were carried out by independent investigators blinded to the treatment condition. The surgeon was blinded to all assessments.

**The long-term toxicity test of IMRCs in cynomolgus monkeys**

Cynomolgus monkeys (3-5 years old) received IMRCs through intravenous infusion over 6 months, with injections administered every 1-3 weeks for a total of 22 doses within a 24-week period. Subsequently, the monkeys were monitored for over 12 weeks. Eighteen cynomolgus monkeys (9 females and 9 males) were divided into three groups: low-dose group (2.6 × 10^6^ cells/kg) and high-dose group (2 × 10^7^ cells/kg), or 10 ml saline. The cynomolgus monkeys were observed regularly for clinical symptoms, ophthalmological examination, weight and food intake, body temperature, blood pressure, electrocardiogram, urine examination, hematology, coagulation function and blood chemistry at the quarantine stage, 1 week after administration and recovery period (once every 4 weeks until 36 weeks). At the end of the recovery period (36 weeks), the autopsies were performed for organ weights measure and pathological examination.

**Follow-up assessment in rabbits**

At the baseline, 1^st^ and 2^nd^ weeks post-surgical, and subsequently 1^st^, 2^nd^, 4^th^, 6^th^, 8^th^ weeks of intra-articular injection of IMRCs/normal saline, general behavioral, and weight assessment were done. Regular observation of the operated knee and the IMRCs intra-articular injection area were selected to assess safety and toxicity. Any swelling, redness, color changes, or changes in skin integrity were documented. Systemic safety and toxicity were assessed, including body weight, complete blood count, behavioral assessment (food intake, vocalization, mobility), organ weight, histological examinations and FISH. A complete blood count (CBC) was conducted at week 8 to assess the deviation of several blood parameters from normal in order to investigate hematological or systemic toxicity. The major organs, kidneys, heart, lung, liver and spleen, were weighed after harvesting to examine if there was an increase or decrease in organ weight, which could indicate IMRCs-related organ toxicity. Histopathological analysis and FISH of those major organs were performed to further exploration of IMRCs organ toxicity. A full protocol for H&E staining and FISH analysis was provided in the histological staining section.

**Complete blood count in rabbits**

Complete blood counts were performed at week 8, and all blood parameters’ deviations from the normal values were assessed to investigate toxicity. Under general anesthesia with isoflurane, 2 ml of blood was drawn from the dorsal marginal vein of the lateral ear and transferred to a 2 ml EDTA tube (Becton, 367841). The blood samples were then tested using a fully automatic biochemical analyzer (Shenzhen Ledu Life Technology, Chemray 800).

**Participants eligibility in phase-I clinical trial**

**Inclusion criteria**

Patients are eligible to be included in the clinical trial if they meet all of the conditions below:

1. 18 to 65 years old.
2. Grade Ⅰ-Ⅱ meniscus injury according to the stoller classification standard in MRI.
3. The patients had pain or knee function impairment after accepting three months of nonoperative treatment.

**Exclusion criteria**

Patients should be excluded from the clinical trial if they meet one of the following conditions:

1. Recent history of lower limb fractures or intra-articular injection.
2. The patients who have meniscus injury need the surgical operation.
3. Those aged > 65 years or < 18 years, or patients without complete civil capacity.
4. The patients who have the severe coagulation disorders, cardiopulmonary failure, etc.
5. The women who are pregnant or nursing.
6. There are electronic implants such as pacemakers in the body.
7. The patients with HIV, hepatitis or syphilis infection.
8. The patients who are alcoholism or drug user.
9. The patients who have vestibular and balance disorders.
10. The patients with severe cognitive impairment who cannot follow instructions to complete the treatment.
11. The patients who do not sign the informed consent.
12. The tumor patients.
13. Immunodeficiency patients.
14. Congenital or acquired knee joint deformity.
15. Patients who are participating in other clinical trials or exist other reasons that researchers believe inappropriate for this trial.

**Clinical intervention and IMRCs intra-articular injection**

The process of IMRCs Injection was as follows:

(a) sterile skin preparation, (b) local anesthesia using 2% lidocaine, (c) use of the 22G needle to enter the skin accurately and smoothly, (d) after a sense of breakthrough to confirm entered the knee joint cavity, injected the 3ml different dosage (1 × 10^7^/3 ml, 5 × 10^7^/3 ml, and 1 × 10^8^/3 ml) of IMRCs slowly, (e) pull out the puncture needle, and press for 3-5 minutes to stop the bleeding, (f) disinfect and dress the injected area.

**Implementation and evaluation of meniscal MRI in eighteen participants**

MRI was performed using a 3.0-T scanner (UNITED IMAGING, Achieva 3.0-T) with an 8-channel knee coil. The maximum gradient strength was 80 mT/m, and the maximum slew rate was 200 mT/m/ms. The images were transferred digitally to a picture archiving and communication system. The MRI scan protocol included routine morphological knee sequences of T1-weighted sequences in coronal; T2-weighted sequences with fat saturation (FS) in sagittal, coronal, and axial plane. PD–weighted sequences with FS turbo spin-echo (TSE) in sagittal, coronal. The detailed sequence parameters are displayed in Supplementary Table 10.

The meniscal volumes were calculated using the following methods: We used 3D Slicer software (software version: 4.10.2, <http://www.slicer.org)>. We imported the DICOM format pictures of the sagittal PD fat saturation (FS) turbo spin-echo (TSE) MRI sequences into the 3DSlicer software system. In the “segment editor” mode, drew the meniscus layer by layer, and then generate a 3D meniscus body by “Show 3D” function and finally get the meniscal volume by Labelmap Statistics in the “segment statistics” mode. One independent radiologist calculated the meniscus volume of all participants. The radiologists were all blinded to the time point and the dose of IMRCs administered. The MRI scan was performed at baseline, week 1, week 4, week 8, week 12, and week 48.

**
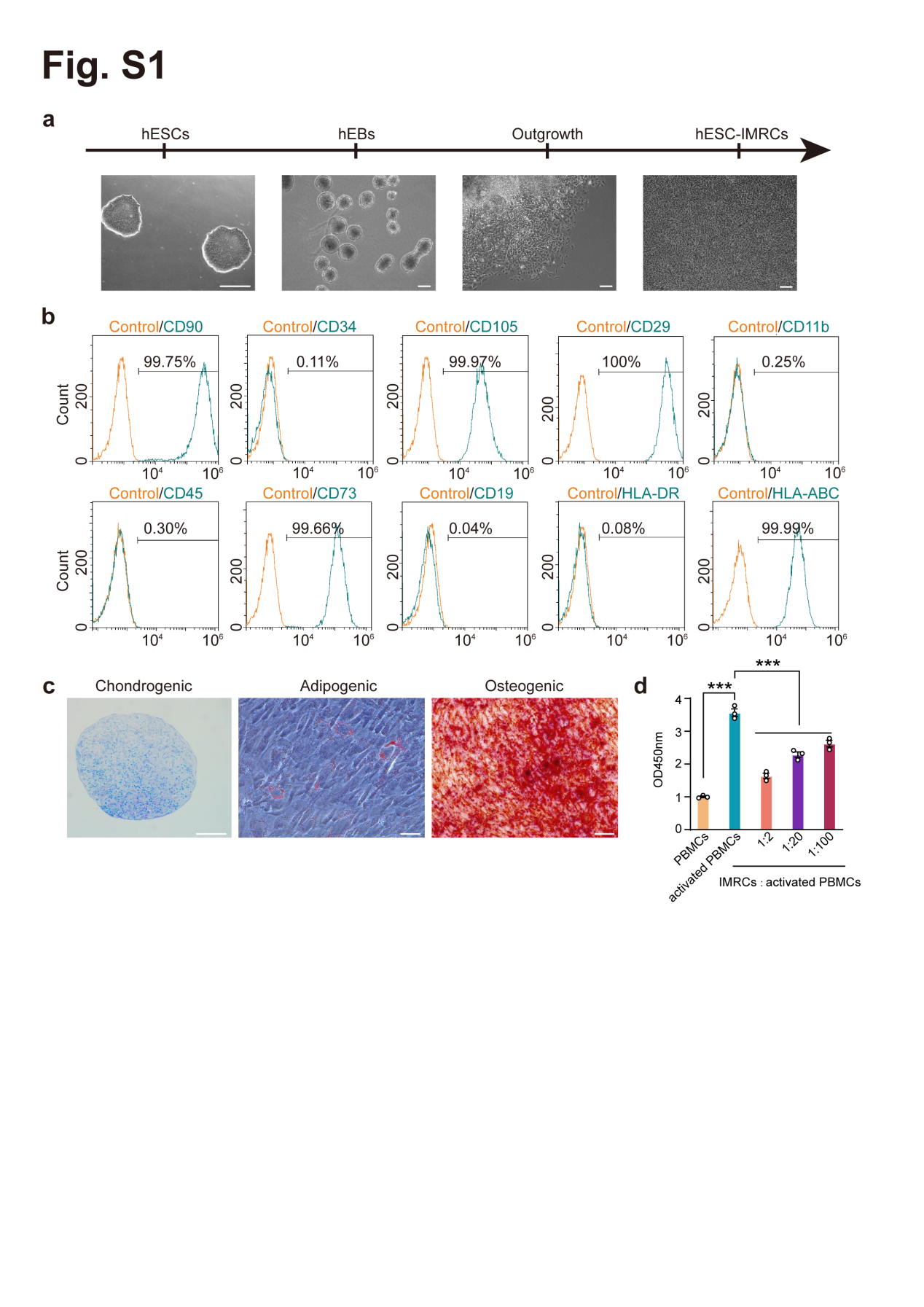
Fig. S1** Characteristics of hESCs-derived-IMRCs. **a** Typical cell morphology during hESCs-derived-IMRCs differentiation. Scale bar, 200 µm. **b** IMRCs’ expression of MSC-specific surface markers was determined by flow cytometry. **c** Multipotency of hESCs-IMRCs was examined by differentiation into chondrogenic (Alcian Blue staining), adipogenic (Oil Red O staining), and osteogenic (Alizarin Red staining) lineages. **d** The inhibitory effects of IMRCs on activated PBMC (CD3/28 and IL-2 co-stimulation) proliferation at ratios of 1:2, 1:20, 1:100 and 1:200 in vitro. **P* < 0.05, ***P* < 0.01, ***P < 0.001; Data are represented as the mean ± SEM. Scale bar: 100 µm.

**
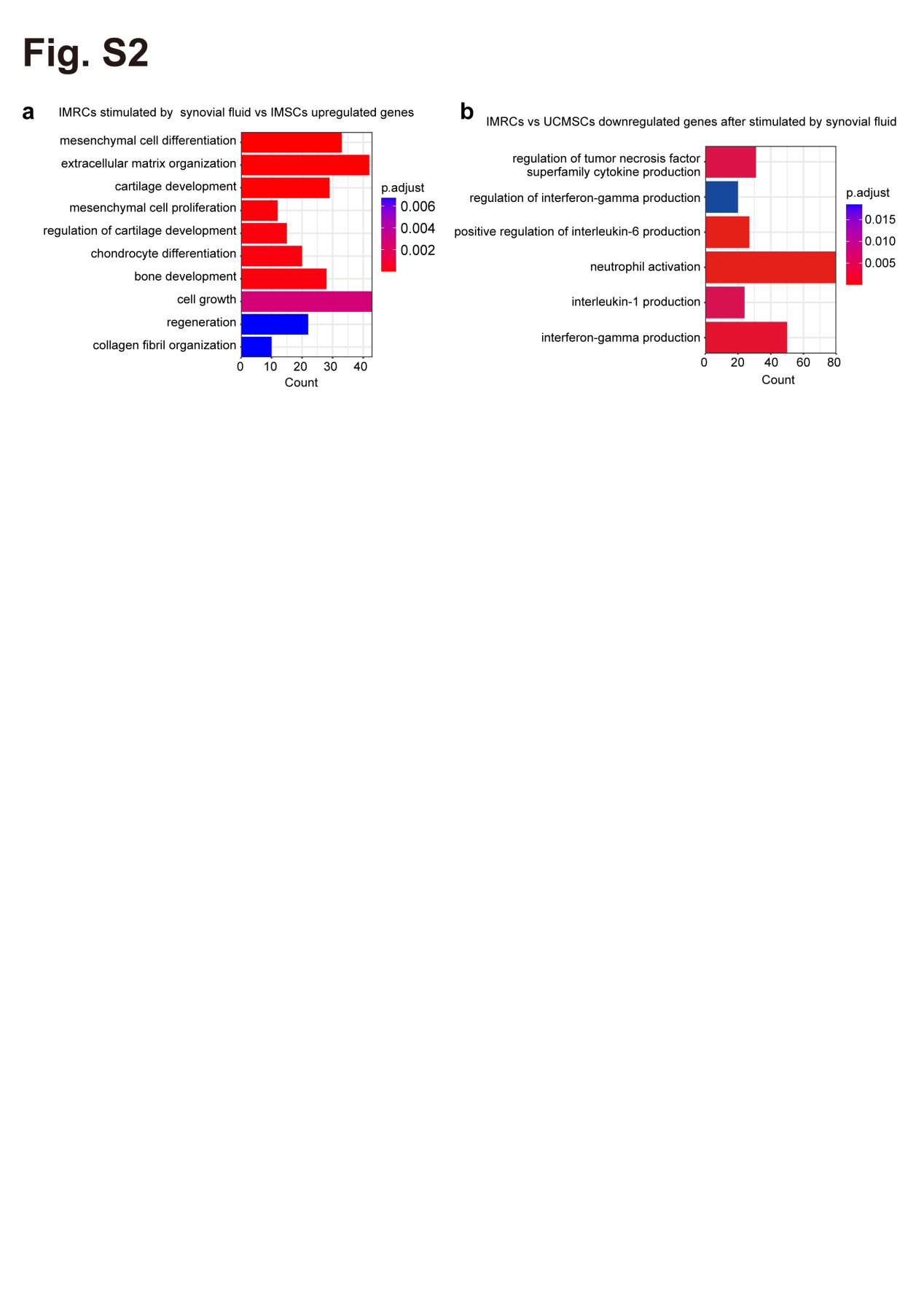
**

**Fig. S2** GO analysis of IMRCs versus UCMSCs after synovial fluid stimulation. **a** GO biological process (GOBP) analysis of differentially upregulated genes for synovial fluid stimulated IMRCs versus IMRCs. **b** GO biological process (GOBP) analysis of differentially downregulated genes for IMRCs versus UCMSCs after synovial fluid treatment.


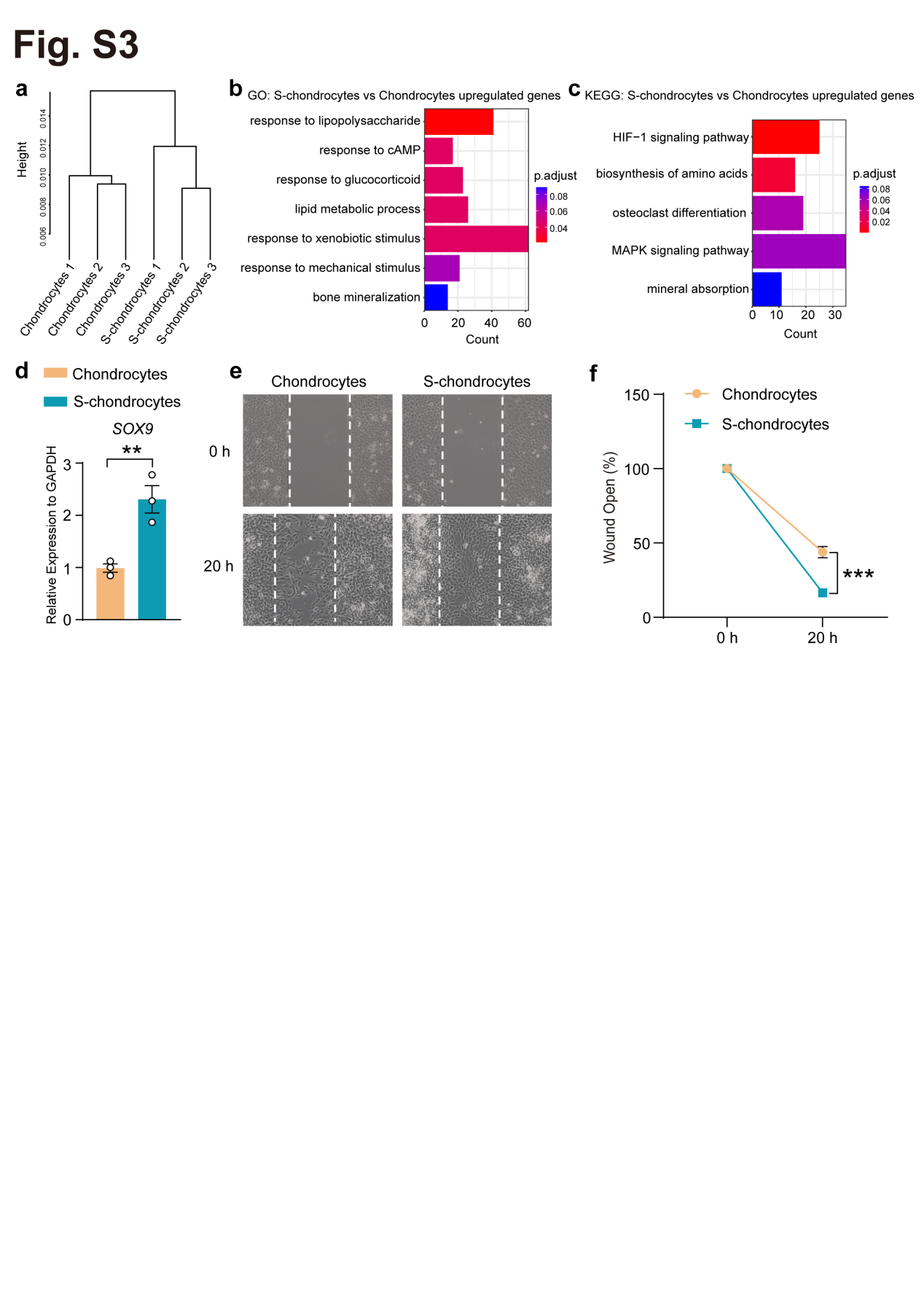


**Fig. S3** IMRCs-conditioned medium co-culture with rat chondrocytes. **a** Unsupervised hierarchical clustering analysis of chondrocytes before and after IMRCs-conditioned medium stimulation (S-chondrocytes). **b** GO biological process (GOBP) analysis of differentially upregulated genes for S-chondrocytes versus chondrocytes. **c** KEGG analysis of differentially upregulated genes for S-chondrocytes versus chondrocytes. **d** Real-Time PCR (RT-PCR) analysis of *SOX9* genes. **e** Pictures of cell morphology before and after IMRCs-conditioned medium co-culture. **f** Statistics on the wound area in (d). **P* < 0.05, ***P* < 0.01, ****P* < 0.001; Data are represented as the mean ± SEM.


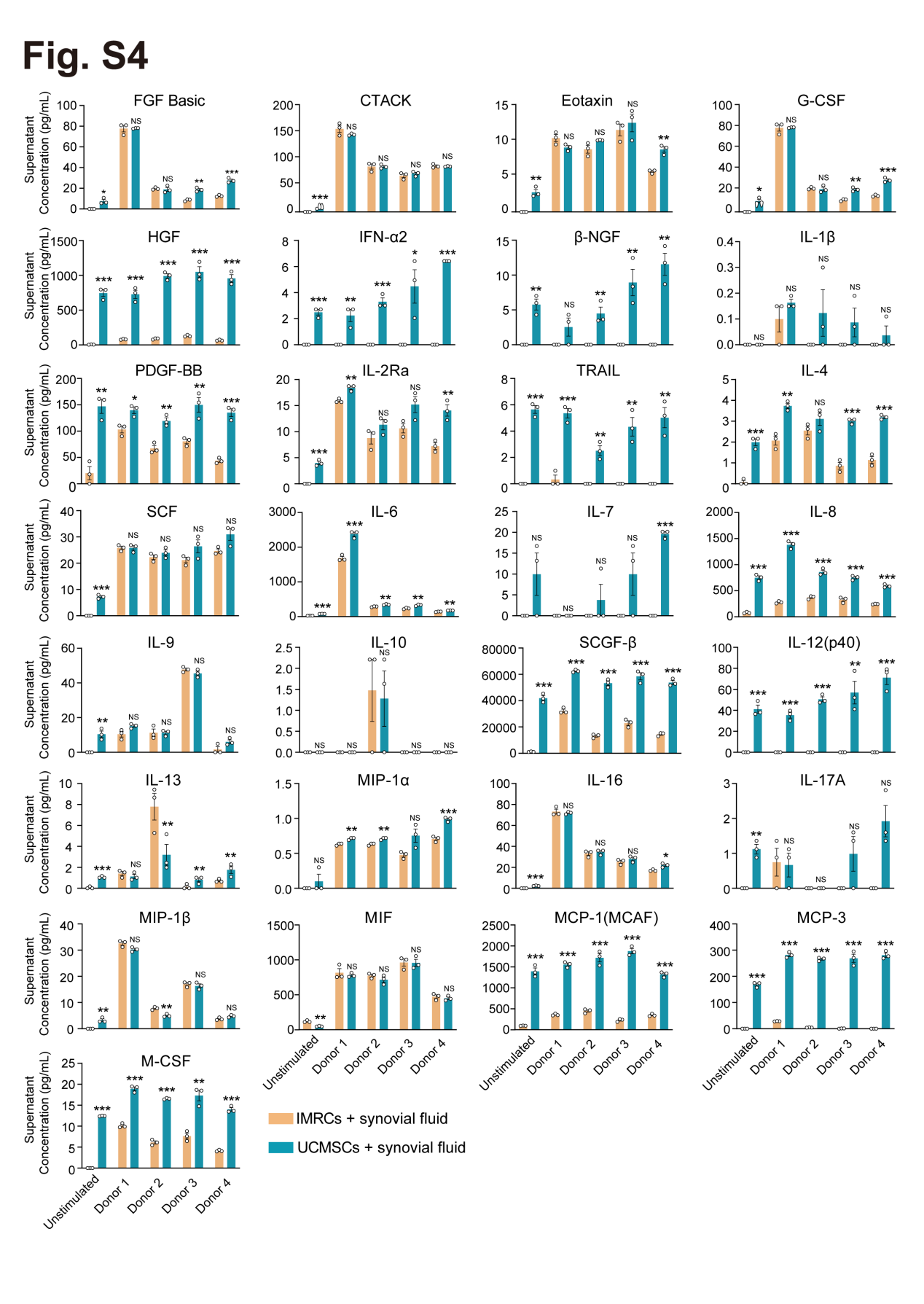


**Fig. S4** Cytokines detection of IMRCs and UCMSCs in the supernatant. ELISA analysis of biologically relevant chemokines and cytokines in the supernatant of IMRCs and UCMSCs with and without synovial fluid stimulation. **P* < 0.05, ***P* < 0.01, ****P* < 0.001, NS, not significant; Data are represented as the mean ± SEM.


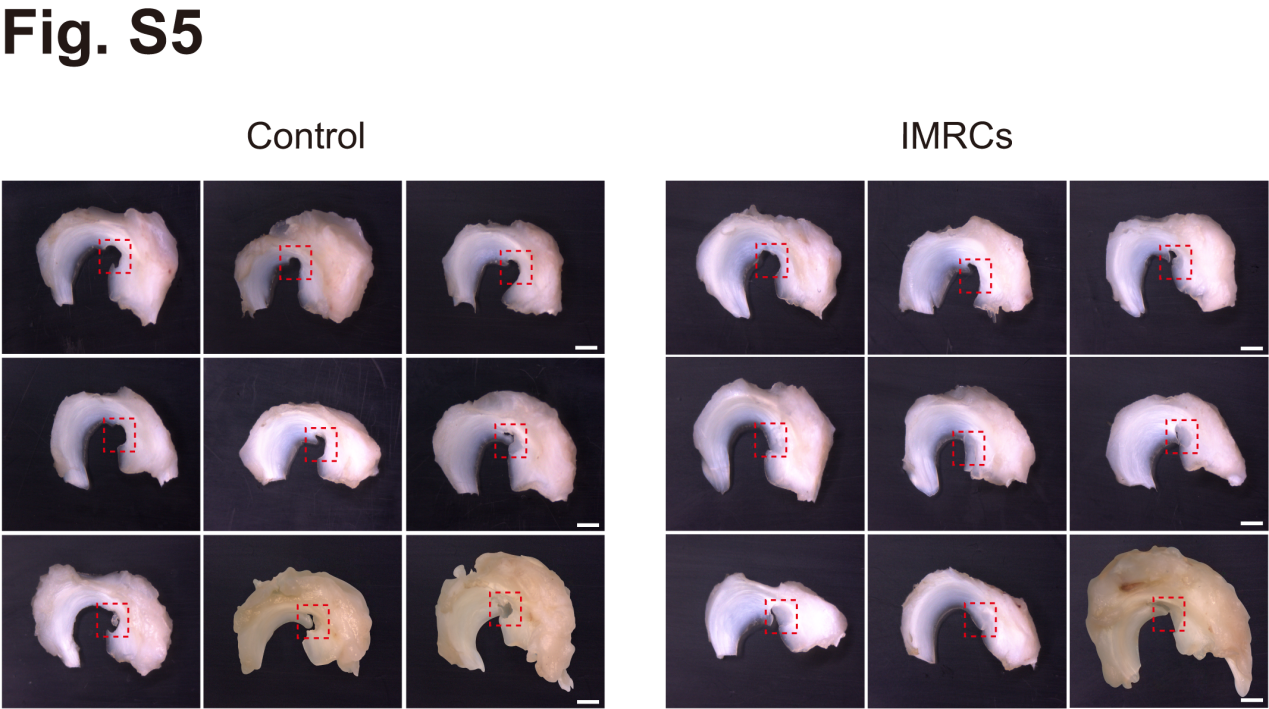


**Fig. S5** Macroscopic observation of the meniscus of all rabbits at 8 weeks after IMRCs implantation. The red boxes show the repair of defective lesions in the meniscus. n = 9 in each group. Scale bar: 2 mm.


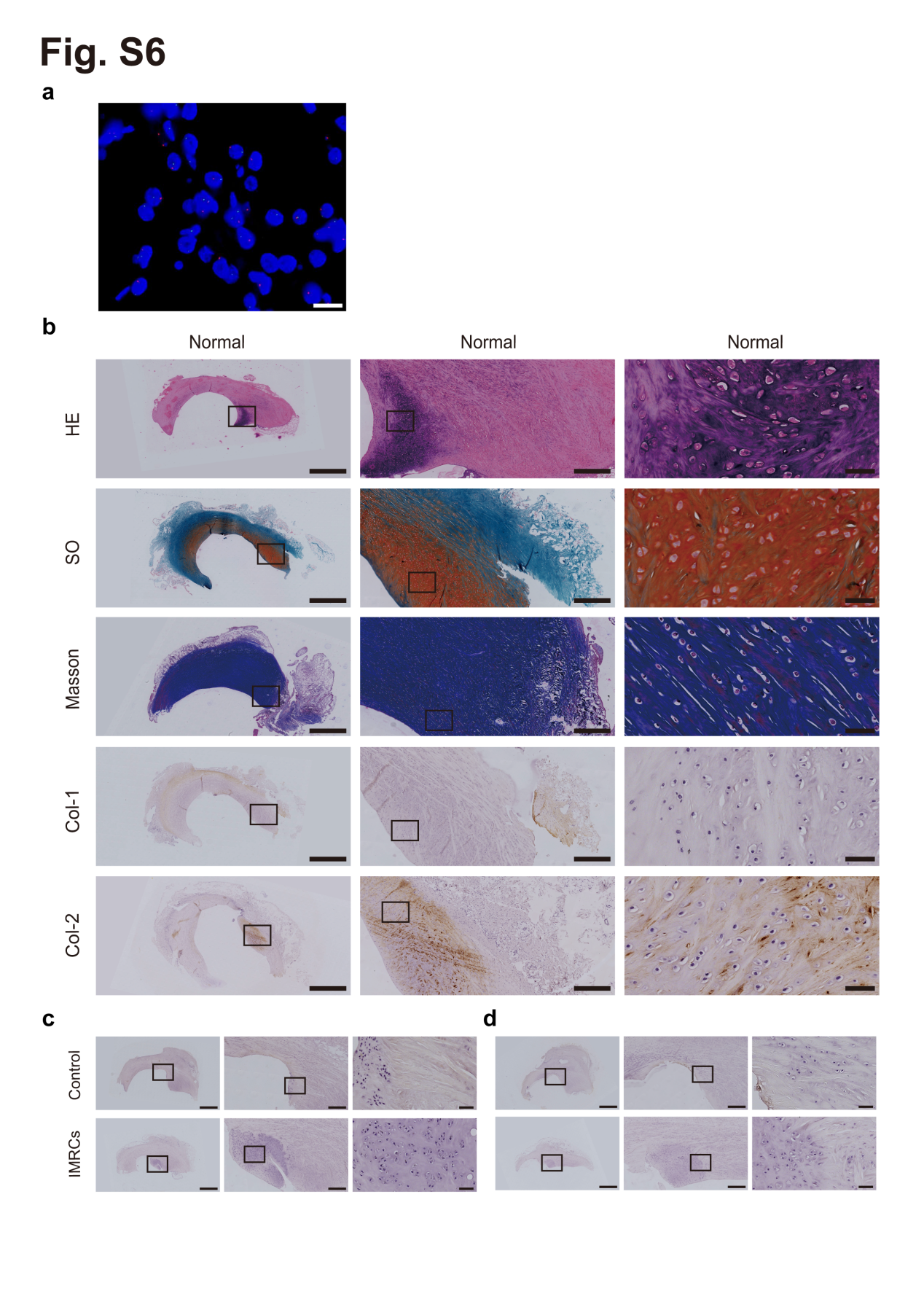


**Fig. S6** Positive control of FISH analysis, Histological staining of rabbit normal meniscus and negative control for IHC staining. **a** FISH analysis of human DNA-specific probes. The green arrow represents the human X-chromosome mitoses (Xp11.1-q11.1) and the red arrow represents the human Y-chromosome trophoblast (Yp11.1-q11.1). Blue cells pointed by the blue arrows represent DAPI (4',6-diamidino-2-phenylindole) fluorescent stain-colored nuclei. Scale bar: 20 μm. **b** SO, Masson, Col-1, Col-2 staining of normal meniscus in rabbits. **c, d** Col-1, Col-2 immunohistochemical negative control staining. The boxed areas are shown at higher magnification. For **b-d**, Scale bar: 2.5 mm, 500 μm, 50 μm.


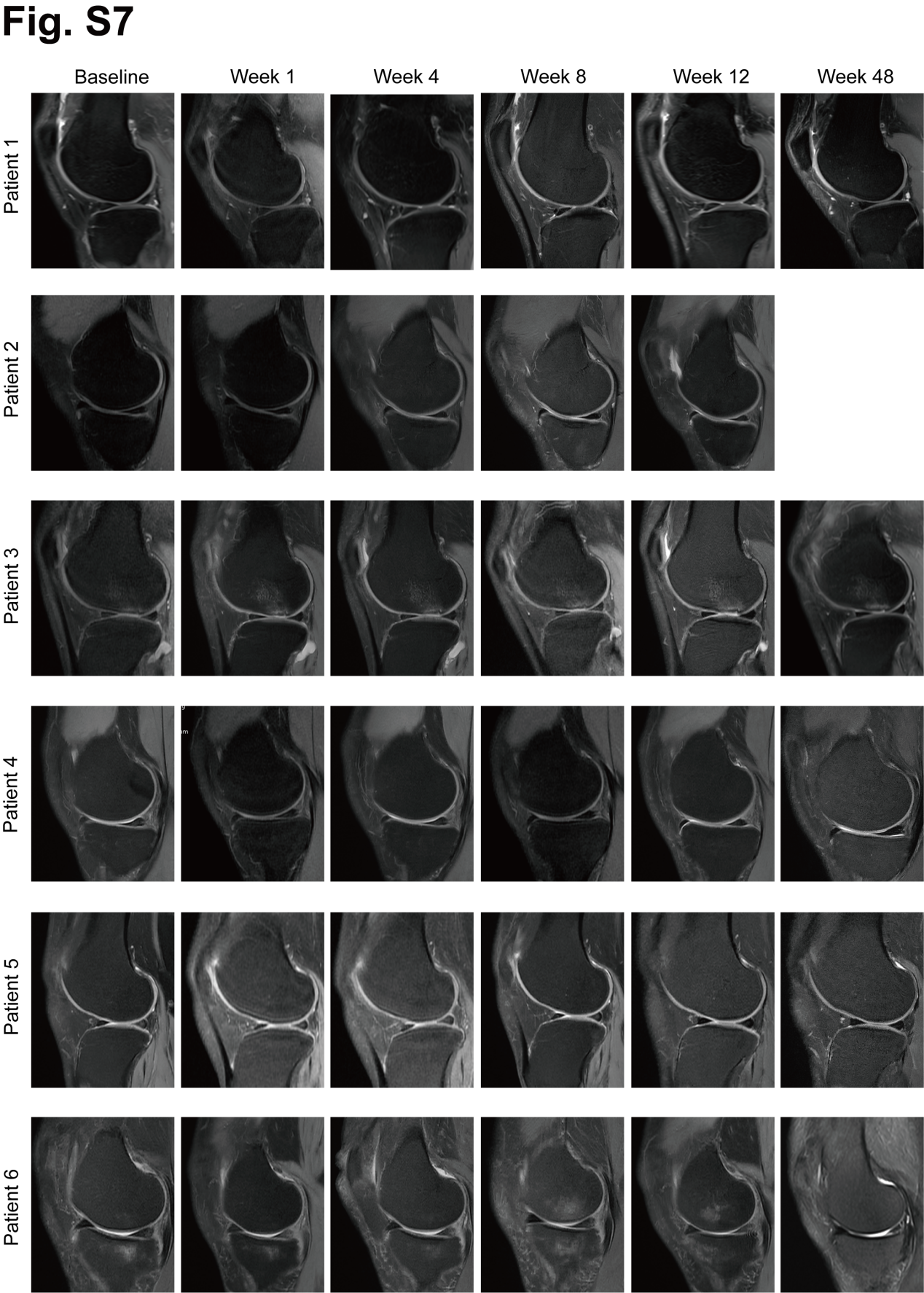


**Fig. S7** The sagittal view MR images of patients in the low-dose group.


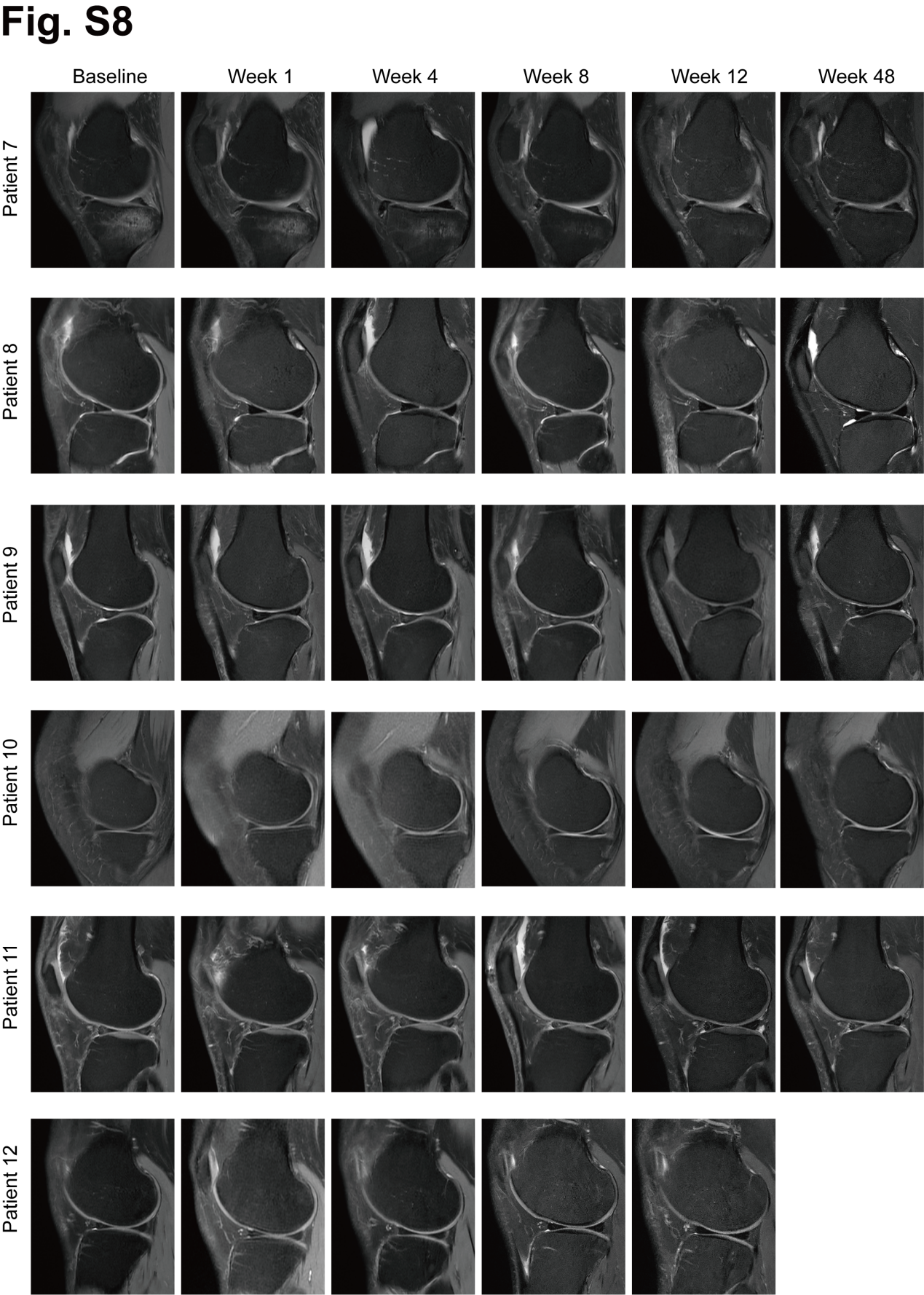


**Fig. S8** The sagittal view MR images of patients in the mid-dose group.


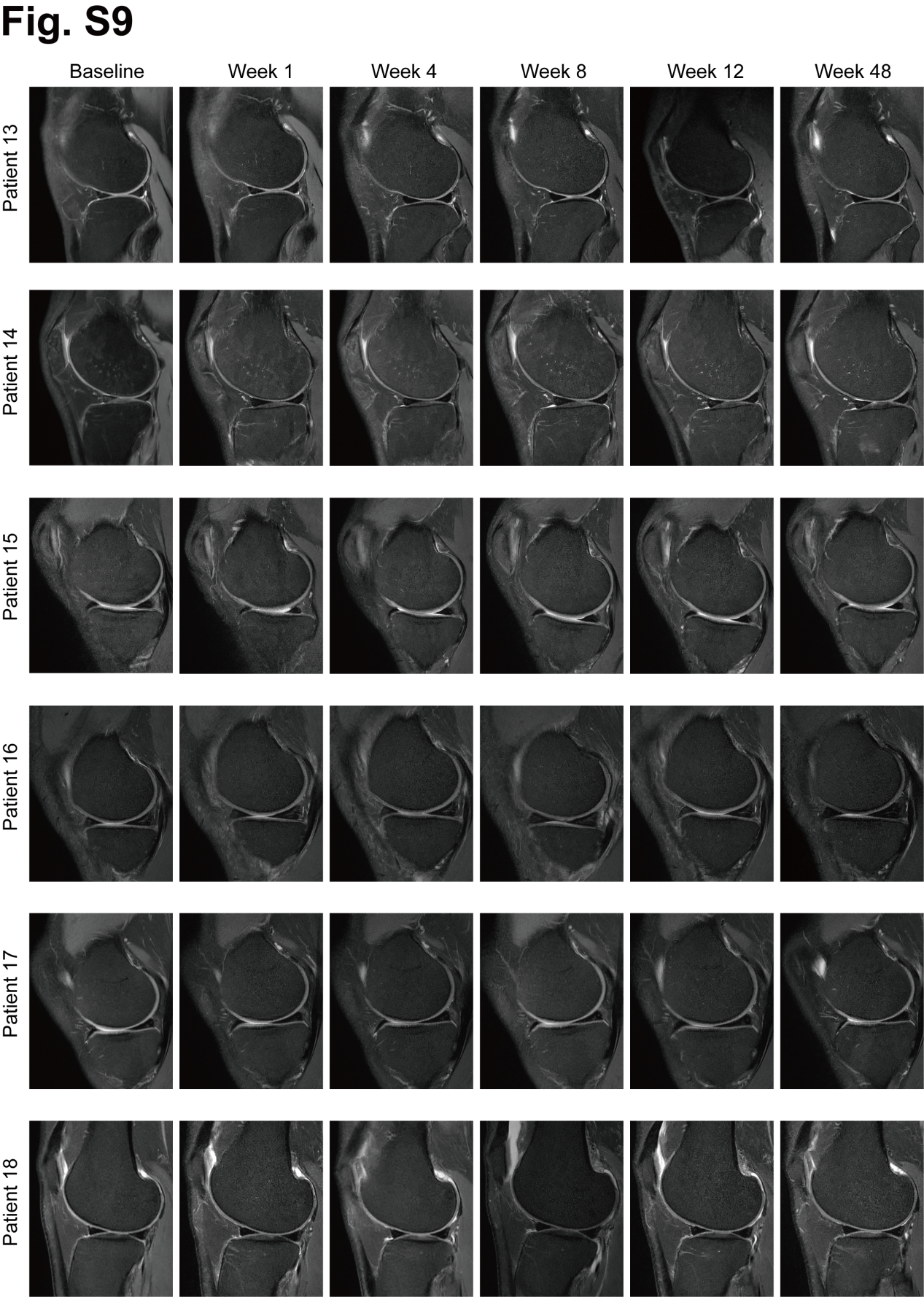


**Fig. S9** The sagittal view MR images of patients in the high-dose group.

**Table S1. The [characteristics](javascript:;) of the 4 donors in the synovial fluid analysis.**

|  | **Donor 1** | **Donor 2** | **Donor 3** | **Donor 4** |
| --- | --- | --- | --- | --- |
| Date | 2022.5.9 | 2022.5.19 | 2022.6.1 | 2022.6.7 |
| Age (years) | 60 | 60 | 57 | 57 |
| Gender | Female | Female | Female | Female |
| Knee laterality | Left | Left | Right | Right |
| Main symptoms | Knee joint pain, swelling | Knee joint pain, swelling | knee joint pain, swelling | knee joint pain, swelling |
| Duration from symptoms onset (days) | 32 | 5 | 3 | 13 |
| MR images of knee | Meniscus torn, laceration of ACL, fluid swelling in knee cavity | Meniscus torn, fluid swelling in knee cavity | Meniscus injury and incomplete laceration of ACL, PCL and MCL. Fluid swelling in knee cavity | Meniscus injury, tibial bone edema, injured ACL, MCL and MPR. Fluid swelling in the knee cavity |
| Feature of synovial fluid | Reddish | Reddish | Reddish | Reddish |
| Volume of synovial fluid (mL) | 20 | 12 | 8 | 20 |

Abbreviations: ACL, anterior cruciate ligament; PCL, posterior cruciate ligament; MCL, medial collateral ligament; MPR, medial patellar retinaculum.

Table S2 (Separate file).

GO list of IMRCs vs UCMSCs upregulated genes after stimulated by synovial fluid.

**Table S3. Cytokines analysis of donors’ synovial fluid.**

| **Cytokines**  (pg/mL) | **Donor 1** | **Donor 2** | **Donor 3** | **Donor 4** |
| --- | --- | --- | --- | --- |
| CTACK | 41.7±13.99 | 13.93±5.78 | 0.79±0.41 | 163.51±6.28 |
| Eotaxin | 9.15±0.50 | 0.12±0.12 | 5.35±1.77 | 5.05±0.46 |
| Basic FGF | 14.41±0.31 | 8.34±0.52 | 9.88±0.74 | 11.21±0.42 |
| G-CSF | 313.86±16.34 | 49.58±4.24 | 74.77±15.5 | 94.03±6.36 |
| GM-CSF | ND | ND | ND | ND |
| GRO-α | ND | ND | ND | ND |
| HGF | 123.05±7.89 | 457.04±35.76 | 1578.91±418.88 | 612.70±44.05 |
| IFN-α2 | ND | ND | ND | ND |
| IFN-γ | ND | ND | ND | ND |
| IL-1α | 7.21±1.67 | ND | ND | 0.41±0.41 |
| IL-1β | 0.24±0.02 | 0.22±0.09 | 0.38±0.16 | 0.64±0.04 |
| IL-1RA | 3814.93±285.77 | 3963.82±1343.29 | 3943.11±254.76 | 3552.40±28.13 |
| IL-2 | ND | ND | ND | ND |
| IL-2Ra | 26.08±0.97 | ND | ND | ND |
| IL-3 | ND | ND | ND | ND |
| IL-4 | 1.82±0.09 | 0.67±0.23 | 1.20±0.19 | 1.04±0 |
| IL-5 | ND | ND | ND | ND |
| IL-6 | 542.01±79.32 | 49.29±30.48 | 14.94±2.66 | ND |
| IL-7 | ND | ND | ND | ND |
| IL-8 | 157.98±18.03 | 20.44±5.68 | 25.98±6.10 | 14.49±0.32 |
| IL-9 | 6.66±1.08 | ND | 4.46±4.46 | ND |
| IL-10 | 5.46±0.32 | ND | 1.85±0.69 | ND |
| IL-12 (p70) | ND | ND | ND | ND |
| IL-12 (p40) | 56.82±2.54 | 75.04±3.54 | 128.11±28.29 | 120.04±8.09 |
| IL-13 | 0.25±0.04 | ND | 0.07±0.07 | ND |
| IL-15 | ND | ND | ND | ND |
| IL-16 | 152.11±7.40 | 26.55±3.55 | 15.29±3.82 | 17.27±0.61 |
| IL-17A | 0.64±0.64 | ND | ND | ND |
| IL-18 | ND | ND | ND | ND |
| IP-10 | 210.63±15.81 | 109.47±37.06 | 128.43±27.49 | 208.98±9.82 |
| LIF | 24.06±3.55 | 2.19±2.19 | 17.19±2.91 | 8.08±1.24 |
| MCP-1 | 494.25±14.53 | 359.33±113.25 | 22.97±5.54 | 20.19±1.11 |
| MCP-3 | 23.61±0.82 | ND | ND | ND |
| M-CSF | 3.09±0.06 | 0.94±0.94 | ND | ND |
| MIF | 370.65±30.91 | 91.25±46.59 | 128.51±25.54 | 100.73±6.36 |
| MIG | 351.86±7.99 | 108.04±4.38 | 81.03±17.38 | 202.77±4.05 |
| MIP-1α | 20.50±0.51 | 1.48±0.28 | 3.47±0.54 | 3.60±0.05 |
| MIP-1β | 216.2±5.42 | 5.09±1.13 | 22.78±4.93 | 8.29±0.46 |
| β-NGF | ND | ND | ND | ND |
| PDGF-BB | 23.25±5.69 | 2.60±2.60 | 36.17±12.68 | 10.43±0.92 |
| RANTES | ND | ND | 135.63±43.97 | ND |
| SCF | 178.87±7.67 | 114.62±20.14 | 158.57±29.02 | 230.58±4.82 |
| SDF-1α | 348.89±8.64 | 250.9±55.45 | 283.22±48.56 | 401.02±29.81 |
| TNF-α | 3.14±0.09 | 2.53±0.98 | 5.74±1.76 | 3.42±0 |
| TNF-β | 5.00±2.03 | ND | 4.72±4.72 | ND |
| TRAIL | 2.44±0.51 | ND | ND | ND |
| VEGF-A | ND | ND | ND | ND |

ND, not detection; Every donor was tested three times. Data are represented as mean ± SEM.

**Table S4. Complete blood counts of rabbits at week 8.**

| **Variables** | **Control group** | **IMRCs group** | **Reference range** |
| --- | --- | --- | --- |
| WBC (10^9^/L) | 8.42±1.01 | 9.24±1.02 | 5.20-13.5 |
| Lymphocytes (10^9^/L) | 3.14±0.39 | 3.53±0.33 | 3.20-9.0 |
| Monocytes (10^9^/L) | 0.42±0.05 | 0.39±0.06 | 0.10-0.60 |
| Granulocytes (10^9^/L) | 4.91±0.63 | 6.00±0.82 | 2.0-7.50 |
| Lymph percent (%) | 39.53±1.84 | 32.76±2.86 | 35.20-75.60 |
| Mono percent (%) | 4.95±0.25 | 4.26±0.23 | 2.50-6.00 |
| Gran percent (%) | 55.52±1.79 | 58.70±2.94 | 20.20-59.3 |
| RBC (10^12^/L) | 5.58±0.52 | 6.09±0.18 | 5.00-7.60 |
| HGB (g/L) | 121.79±12.20 | 124.57±4.34 | 105-170 |
| HCT (%) | 37.57±3.46 | 39.30±1.12 | 31-46 |
| MCV (fL) | 66.91±1.02 | 64.66±0.38 | 56.80-66.50 |
| MCH (pg) | 21.45±0.51 | 20.41±0.27 | 20.10-25.10 |
| MCHC (g/L) | 317.64±7.01 | 319.64±2.95 | 320-370 |
| RDW (%) | 13.16±0.25 | 13.39±0.26 | 13.00-18.50 |
| Platelets (10^9^/L) | 518.86±52.47 | 316.00±45.69 | 100-712 |
| MPV (fL) | 6.68±0.12 | 6.47±0.28 | 3.80-6.80 |
| PDW | 16.86±0.13 | 16.56±0.19 | ---------- |
| PCT (%) | 0.35±0.04 | 0.20±0.02 | ---------- |

Abbreviations: WBC, white blood cells; RBC, [red blood cell](javascript:;)s; HGB, [hemoglobin](javascript:;); HCT, hematocrit; MCV, mean corpuscular volume; MCH, mean corpuscular hemoglobin; MCHC, [mean corpuscular hemoglobin concentration](javascript:;); RDW, red blood cells distribution width; MPV, Mean platelet volume; PDW, platelet distribution width; PCT, plateletcrit. Data are presented as mean ± SEM, n = 7 in each group.

**Table S5. Long-term toxicity test in cynomolgus monkeys: blood test.**

|  | **Baseline** | | | **Week 1** | | | **Week 4** | | | **Week 8** | | | **Week 12** | | | **Week 36** | | |
| --- | --- | --- | --- | --- | --- | --- | --- | --- | --- | --- | --- | --- | --- | --- | --- | --- | --- | --- |
|  | **Control**  **(n=6)** | **Low-dose**  **(n=6)** | **High-dose**  **(n=6)** | **Control**  **(n=6)** | **Low-dose**  **(n=6)** | **High-dose**  **(n=6)** | **Control**  **(n=6)** | **Low-dose**  **(n=6)** | **High-dose**  **(n=6)** | **Control(n=6)** | **Low-dose**  **(n=6)** | **High-dose**  **(n=6)** | **Control(n=6)** | **Low-dose**  **(n=6)** | **High-dose**  **(n=6)** | **Control**  **(n=6)** | **Low-dose**  **(n=6)** | **High-dose**  **(n=6)** |
| **WBC**  **(10^9/^L)** | 9.35±3.24 | 9.16±2.90 | 10.48±4.63 | 9.07±2.69 | 10.00±2.14 | 14.60±6.96 | 9.35±0.63 | 9.56±3.31 | 9.97±2.76 | 8.41±1.63 | 9.14±3.05 | 9.77±1.88 | 8.92±1.87 | 9.05±2.06 | 10.84±2.34 | 7.55±0.74 | 7.46±1.10 | 8.64±1.80 |
| **Neut**  **(10^9^/L)** | 4.23±2.44 | 5.51±3.57 | 5.82±4.29 | 5.12±2.91 | 6.88±2.21 | 10.68±6.90 | 3.17±1.31 | 3.94±3.23 | 2.28±0.94 | 2.52±1.32 | 3.67±2.08 | 2.93±1.33 | 2.88±0.74 | 3.14±1.26 | 3.36±1.52 | 1.53±0.44 | 2.47±1.73 | 2.23±0.56 |
| **Lymph**  **(10^9^/L)** | 4.53±0.71 | 3.34±1.19 | 4.29±1.69 | 3.55±0.65 | 2.85±1.47 | 3.57±1.44 | 5.56±1.34 | 5.12±1.96 | 7.17±2.40 | 5.38±1.55 | 5.02±2.01 | 6.37±2.09 | 5.35±1.77 | 5.34±1.49 | 6.87±2.31 | 5.45±1.20 | 4.21±0.57 | 5.82±2.56 |
| **Mono**  **(10^9^/L)** | 0.30±0.14 | 0.17±0.05 | 0.19±0.06 | 0.24±0.09 | 0.15±0.07 | 0.23±0.09 | 0.33±0.05 | 0.23±0.09 | 0.23±0.09 | 0.32±0.03 | 0.22±0.08 | 0.27±0.10 | 0.50±0.30 | 0.24±0.08 | 0.31±0.10 | 0.35±0.06 | 0.28±0.08 | 0.31±0.14 |
| **Eosl**  **(10^9^/L)** | 0.11±0.04 | 0.07±0.05 | 0.10±0.07 | 0.06±0.03 | 0.04±0.03 | 0.02±0.02 | 0.17±0.16 | 0.16±0.09 | 0.18±0.12 | 0.09±0.03 | 0.17±0.16 | 0.11±0.11 | 0.08±0.04 | 0.25±0.16 | 0.19±0.18 | 0.09±0 | 0.41±0.13 | 0.14±0.11 |
| **RBC**  **(10^9^/L)** | 5.55±0.30 | 5.41±0.43 | 5.70±0.44 | 5.13±0.37 | 5.09±0.40 | 5.35±0.48 | 5.49±0.38 | 5.68±0.35 | 5.57±0.43 | 5.64±0.24 | 5.67±0.27 | 6.02±0.39 | 5.72±0.33 | 5.75±0.43 | 6.03±0.59 | 5.05±0.01 | 5.33±0.77 | 5.66±0.64 |
| **Retic**  **(10^9^/L)** | 56.74±14.36 | 70.18±10.59 | 67.45±15.26 | 133.70±32.60 | 145.70±33.00 | 140.80±66.90 | 70.32±12.46 | 71.65±18.11 | 86.28±31.25 | 69.88±18.60 | 72.43±21.38 | 80.68±27.63 | 64.98±14.03 | 67.25±24.37 | 84.77±29.84 | 52.85±28.50 | 91.25±2.76 | 78.70±12.73 |
| **HGB**  **(g/L)** | 132.28±9.69 | 129.33±11.59 | 137.00±8.27 | 153.00±4.00 | 156.00±9.00 | 159.00±5.00 | 131.00±12.02 | 135.00±8.34 | 137.33±88.64 | 133.17±12.60 | 134.50±7.42 | 141.83±7.96 | 132.50±14.10 | 135.00±11.20 | 141.33±11.70 | 122.50±9.19 | 130.50±17.60 | 136.50±12.60 |
| **PLT**  **(10^9^/L)** | 409.22±28.96 | 438.17±107.09 | 338.33±69.81 | 1055.00±160.00 | 1096.00±155.00 | 1089.00±196.00 | 389.33±34.67 | 409.67±89.39 | 327.67±81.34 | 382.33±39.71 | 385.00±86.02 | 343.33±70.23 | 338.67±108.72 | 417.33±101.34 | 321.17±53.35 | 303.00±1.41 | 302.00±127.8 | 256.00±70.71 |
| **ALT (U/L)** | 52.50±28.03 | 52.33±22.85 | 58.17±20.68 | 54.33±26.54 | 55.83±26.04 | 63.33±27.23 | 53.40±24.18 | 48.80±14.34 | 51.20±15.61 | 48.00±34.38 | 41.17±16.51 | 75.50±69.44 | 47.50±22.26 | 48.83±22.17 | 52.50±35.66 | 70.50±12.02 | 48.50±13.44 | 121.00±29.70 |
| **AST (U/L)** | 40.83±9.85 | 44.17±20.16 | 41.00±6.42 | 48.67±7.66 | 44.17±17.93 | 55.17±16.38 | 39.40±8.91 | 37.20±5.97 | 39.80±10.38 | 39.00±9.38 | 36.67±13.62 | 43.00±10.88 | 35.50±3.39 | 43.00±20.54 | 38.17±6.71 | 63.50±2.12 | 46.50±34.65 | 77.00±29.70 |
| **TP (g/L)** | 79.23±3.26 | 77.85±3.60 | 73.85±5.75 | 79.62±1.05 | 77.32±2.17 | 72.42±5.08 | 79.02±3.17 | 76.60±4.83 | 72.98±6.76 | 79.27±1.84 | 78.48±1.81 | 75.77±4.05 | 81.15±5.25 | 79.78±4.28 | 76.87±5.32 | 79.30±0.71 | 76.10±4.95 | 78.30±0.14 |
| **ALB (g/L)** | 43.60±3.91 | 45.68±3.54 | 43.25±3.03 | 44.37±3.51 | 45.82±2.99 | 43.60±2.28 | 42.38±3.39 | 43.54±3.95 | 41.50±4.02 | 43.83±2.91 | 45.12±2.90 | 43.13±1.74 | 44.60±4.37 | 45.70±4.81 | 44.00±2.90 | 42.75±3.18 | 43.10±2.97 | 45.05±0.64 |
| **A/G** | 1.23±0.18 | 1.42±0.14 | 1.42±0.07 | 1.28±0.20 | 1.46±0.14 | 1.52±0.13 | 1.16±0.12 | 1.32±0.11 | 1.32±0.09 | 1.25±0.15 | 1.37±0.20 | 1.33±0.07 | 1.22±0.12 | 1.35±0.17 | 1.34±0.06 | 1.18±0.16 | 1.31±0.01 | 1.36±0.04 |
| **TBIL**  **(μmol/L)** | 3.93±0.73 | 4.83±0.72 | 5.33±2.38 | 4.66±0.49 | 4.72±0.57 | 5.83±2.12 | 4.32±0.94 | 4.45±0.58 | 5.60±2.05 | 3.79±0.60 | 4.34±1.14 | 4.20±1.13 | 3.35±0.70 | 3.85±0.79 | 3.73±1.11 | 5.63±0.62 | 5.45±1.46 | 8.90±2.38 |
| **LDH (U/L)** | 417.17±132.74 | 358.17±81.42 | 492.33±149.82 | 500.67±277.66 | 354.17±111.85 | 483.83±249.61 | 334.20±83.56 | 309.40±71.20 | 321.80±39.31 | 405.67±203.90 | 314.50±67.07 | 363.50±65.22 | 395.50±110.86 | 325.50±64.88 | 358.00±63.97 | 421.00±113.14 | 334.00±60.81 | 441.50±89.80 |
| **ALP (U/L)** | 230.33±104.59 | 243.00±64.62 | 216.00±109.02 | 258.17±91.03 | 254.67±76.15 | 216.67±113.45 | 246.80±78.43 | 221.60±86.64 | 250.20±100.95 | 201.17±92.60 | 210.33±62.02 | 219.50±123.40 | 216.67±94.67 | 234.67±79.95 | 221.00±124.17 | 192.00±107.48 | 221.50±37.48 | 180.00±32.53 |
| **CK (U/L)** | 268.50±193.70 | 194.33±63.19 | 344.83±174.59 | 275.17±89.97 | 178.33±88.53 | 3923.33±1613.62 | 300.00±181.27 | 216.00±55.95 | 260.60±98.43 | 235.17±171.30 | 167.67±82.45 | 219.83±86.22 | 197.33±100.91 | 204.50±85.51 | 209.83±82.62 | 329.00±171.12 | 205.00±120.21 | 563.50±211.42 |
| **GLU**  **(mmol/L)** | 4.26±0.69 | 3.00±0.49 | 3.83±1.42 | 4.14±1.21 | 3.89±0.53 | 2.70±0.69 | 4.91±0.49 | 4.06±0.70 | 3.14±1.30 | 5.02±1.43 | 4.78±1.36 | 4.03±1.60 | 4.08±0.97 | 5.19±1.80 | 4.31±1.47 | 4.36±1.04 | 4.86±0.50 | 2.85±0.34 |
| **TG**  **(mmol/L)** | 0.31±0.16 | 0.23±0.06 | 0.28±0.11 | 0.35±0.21 | 0.30±0.12 | 0.35±0.08 | 0.35±0.22 | 0.32±0.09 | 0.34±0.21 | 0.32±0.13 | 0.27±0.09 | 0.32±0.14 | 0.44±0.17 | 0.30±0.11 | 0.39±0.19 | 0.23±0.08 | 0.20±0.03 | 0.28±0.03 |
| **CHOL**  **(mmol/L)** | 3.58±0.63 | 2.95±0.51 | 3.05±0.49 | 3.45±0.52 | 2.75±0.55 | 2.71±0.66 | 3.66±0.43 | 2.97±0.36 | 3.45±0.39 | 3.42±0.51 | 2.82±0.53 | 3.09±0.46 | 3.61±0.40 | 2.98±0.75 | 3.18±0.45 | 3.88±0.16 | 3.42±0.69 | 4.08±0.25 |
| **UREA**  **(mmol/L)** | 7.33±0.83 | 7.70±0.89 | 6.95±1.18 | 6.88±0.93 | 6.65±0.89 | 7.72±1.68 | 5.44±1.34 | 5.50±0.93 | 5.20±0.56 | 6.68±0.69 | 6.32±1.16 | 6.10±1.14 | 6.77±0.68 | 6.13±1.52 | 6.42±1.01 | 8.55±1.48 | 6.90±0 | 8.35±0.49 |
| **CRE**  **(μmol/L)** | 76.00±22.57 | 78.00±13.75 | 79.83±20.41 | 77.50±23.64 | 75.83±12.45 | 88.33±27.38 | 65.00±15.95 | 71.00±14.83 | 79.60±21.67 | 67.50±14.64 | 72.17±15.52 | 77.67±21.58 | 73.17±18.89 | 77.67±16.87 | 80.33±23.97 | 80.50±21.92 | 92.00±25.46 | 88.50±27.58 |
| **PT (s)** | 11.83±1.26 | 12.13±1.33 | 11.48±1.04 | 8.40±0.40 | 8.20±0.30 | 8.50±0.20 | 9.45±0.51 | 9.78±0.70 | 9.33±0.56 | 9.08±0.55 | 9.43±0.61 | 8.90±0.64 | 9.75±0.48 | 9.95±0.49 | 9.77±0.89 | 9.20±0.14 | 9.25±0.35 | 9.25±0.92 |
| **APTT (s)** | 16.41±1.73 | 16.85±1.39 | 16.32±1.22 | 13.70±1.10 | 13.10±2.30 | 14.00±1.10 | 16.78±1.84 | 16.82±1.69 | 16.30±1.52 | 16.40±2.08 | 16.63±1.20 | 16.38±1.55 | 16.28±1.44 | 16.52±1.50 | 15.92±1.44 | 17.30±0.28 | 17.50±2.97 | 14.05±0.49 |
| **IgG (g/L)** | 8.87±1.33 | 7.80±0.50 | 7.32±0.92 | 8.47±1.01 | 7.54±0.42 | 6.77±1.20 | 9.20±0.95 | 8.10±0.78 | 7.13±1.41 | 10.01±0.27 | 9.11±0.87 | 8.50±1.21 | 10.40±1.82 | 9.38±0.77 | 8.73±1.68 | 11.37±1.35 | 9.14±0.88 | 8.71±0.06 |
| **IgA (g/L)** | 1.88±0.49 | 1.61±0.58 | 1.65±0.53 | 1.46±0.48 | 1.46±0.48 | 1.53±0.43 | 1.29±0.52 | 1.27±0.47 | 1.40±0.29 | 1.43±0.50 | 1.41±0.49 | 1.70±0.43 | 1.68±0.46 | 1.60±0.56 | 1.77±0.53 | 1.76±0.76 | 2.38±0.59 | 1.79±0.94 |
| **IgM (g/L)** | 0.78±0.48 | 0.61±0.36 | 0.64±0.38 | 0.74±0.47 | 0.62±0.34 | 0.64±0.32 | 0.82±0.49 | 0.88±0.40 | 0.67±0.29 | 0.80±0.51 | 0.76±0.45 | 0.87±0.39 | 0.80±0.50 | 0.81±0.50 | 0.76±0.37 | 0.84±0.46 | 0.40±0.11 | 0.47±0.21 |
| **C3 (g/L)** | 1.15±0.11 | 1.09±0.08 | 1.10±0.10 | 1.18±0.12 | 1.09±0.10 | 1.05±0.11 | 1.17±0.13 | 1.07±0.09 | 1.13±0.07 | 1.13±0.10 | 1.10±0.15 | 1.16±0.08 | 1.18±0.06 | 1.05±0.15 | 1.14±0.08 | 1.08±0.19 | 0.97±0.03 | 1.10±0 |
| **C4 (g/L)** | 0.16±0.03 | 0.18±0.03 | 0.16±0.03 | 0.16±0.03 | 0.18±0.03 | 0.16±0.03 | 0.13±0.02 | 0.15±0.03 | 0.14±0.03 | 0.10±0.03 | 0.12±0.04 | 0.12±0.03 | 0.11±0.02 | 0.12±0.04 | 0.11±0.04 | 0.11±0.03 | 0.15±0.06 | 0.12±0.02 |
| **K+**  **(mmol/L)** | 4.25±0.36 | 4.09±0.27 | 4.25±0.51 | 4.12±0.30 | 3.86±0.31 | 4.48±0.54 | 4.43±0.52 | 4.33±0.61 | 4.63±0.36 | 4.48±0.36 | 4.32±0.33 | 4.60±0.27 | 4.94±0.83 | 4.98±0.27 | 5.20±0.44 | 3.60±0.14 | 4.14±0.18 | 3.99±0.39 |
| **Na+**  **(mmol/L)** | 151.20±2.85 | 150.02±1.68 | 149.42±1.80 | 149.52±2.12 | 148.55±1.58 | 149.87±2.30 | 148.43±2.36 | 148.80±3.04 | 148.62±2.49 | 148.80±1.87 | 149.02±2.48 | 148.83±2.20 | 151.18±3.16 | 151.43±4.18 | 151.72±3.08 | 147.75±0.07 | 148.10±0.85 | 150.15±3.32 |
| **Cl^-^**  **(mmol/L)** | 98.08±1.24 | 99.25±2.29 | 99.63±2.08 | 101.73±0.99 | 103.13±2.78 | 103.57±4.38 | 108.40±1.17 | 109.45±2.60 | 108.78±3.13 | 107.72±1.60 | 109.62±3.30 | 109.12±4.58 | 100.85±1.70 | 101.97±3.97 | 102.20±2.57 | 102.65±0.49 | 105.65±1.34 | 103.55±0.49 |

Abbreviations: WBC, white blood cells; RBC, [red blood cell](javascript:;)s; HGB, [hemoglobin](javascript:;); PLT, platelet; ALT, alanine aminotransferase; AST, aspartate aminotransferase; TP, total protein; ALB, albumin; A/G, albumin/globulin ratio; TBIL, total bilirubin; LDH, [lactic dehydrogenase](javascript:;); ALP, [alkaline phosphatase](javascript:;); CK, [creatine kinase](javascript:;); GLU, glucose; TG, triglyceride; CHOL, cholesterol; CRE, creatinine; PT, [prothrombin time](javascript:;); APTT, activated partial thromboplastin time. Data are presented as mean ± SEM.

**Table S6. The criteria and score for histological assessment of regenerated meniscus.**

| **I. Surface including lamellar layer:**  **I-I. Femoral side:** | | |
| --- | --- | --- |
|  | **3** | Smooth |
|  | **2** | Slight fibrillation or slightly undulating |
|  | **1** | Moderate fibrillation or markedly undulating |
|  | **0** | Severe fibrillation or disruption |
| **I-II. Tibial side:** | | |
|  | **3** | Smooth |
|  | **2** | Slight fibrillation or slightly undulating |
|  | **1** | Moderate fibrillation or markedly undulating |
|  | **0** | Severe fibrillation or disruption |
| **II. Cellularity of chondrocyte:** | | |
|  | **3** | Normal cell distribution |
|  | **2** | Moderately normal cell distribution |
|  | **1** | Hypercellularity or hypocellularity |
|  | **0** | No chondrocyte |
| **III. Collagen fiber organization:** | | |
|  | **3** | Collagen fibers well organized, no separations or tears |
|  | **2** | Collagen fibers moderately organized, slight separations or tears |
|  | **1** | Collagen fiber unorganized, moderate separations or tears |
|  | **0** | Collagen fiber unorganized, severe separations or tears |
| **IV. Matrix staining (safranin-o):** | | |
|  | **3** | Well stained like normal meniscus |
|  | **2** | Moderately stained |
|  | **1** | Slightly stained |
|  | **0** | Not stained |

**Table S7. Summary of adverse events.**

| **Adverse event category** | | **Total Cohort**  **N (%)** | **Low-dose group**  **N (%)** | **Mid-dose group**  **N (%)** | **High-dose group**  **N (%)** |
| --- | --- | --- | --- | --- | --- |
| Patients with AEs^a^ | | 8 (44.4) | 2 (33.3) | 2 (33.3) | 4 (66.3) |
| Total AEs | | 10 (55.6) | 3 (50.0) | 2 (33.3) | 5 (83.3) |
| Relationship to IMRCs injection | |  |  |  |  |
| Definitely related | | 0 (0) | 0 (0) | 0 (0) | 0 (0) |
| Probably related | | 0 (0) | 0 (0) | 0 (0) | 0 (0) |
| Possibly related | | 4 (22.2) | 0 (0) | 1 (16.7) | 3 (50.0) |
| Not related | | 6 (33.3) | 3 (50.0) | 1 (16.7) | 2 (33.3) |
| Patients with SAEs^b^ | | 1 (5.5) | 1 (16.7) | 0 (0) | 0 (0) |
| Treatment-related | | 0 (0) | 0 (0) | 0 (0) | 0 (0) |
| AEs by grade^c^ | Grade 1 | 10 (55.6) | 3 (50.0) | 2 (33.3) | 5 (83.3) |
|  | Grade 2 | 0 (0) | 0 (0) | 0 (0) | 0 (0) |
|  | Grade 3 | 1 (5.5) | 1 (16.7) | 0 (0) | 0 (0) |
|  | Grade 4 | 0 (0) | 0 (0) | 0 (0) | 0 (0) |
|  | Grade 5 | 0 (0) | 0 (0) | 0 (0) | 0 (0) |
| AEs assessed as possibly related to IMRCs injection | |  |  |  |  |
| Pain after injection | | 1 (5.5) | 0 (0) | 1 (16.7) | 0 (0) |
| Local swelling sensation | | 3 (16.7) | 0 (0) | 0 (0) | 3 (50.0) |

N, number of subjects; %, percentage of subjects; ^a^, the AE is defined as any undesired medical incident that does not necessarily have a cause-and-effect relationship with the treatment; ^b^, An SAE is defined as any undesired medical incident that causes death, life threatening, hospitalization, disability, congenital abnormality, or birth death; ^c^, NCI-CTCAE criteria; Abbreviations: AEs, adverse events; SAEs, serious adverse events; NCI-CTCAE, National Cancer Institute Common Terminology Criteria for Adverse Events.

**Table S****8. The long-term safety evaluation of eighteen participants received IMRCs injection: blood test.**

|  | **Baseline** | | | **Week 1** | | | **Week 4** | | | **Week 8** | | | **Week 12** | | | **Week 48** | | |
| --- | --- | --- | --- | --- | --- | --- | --- | --- | --- | --- | --- | --- | --- | --- | --- | --- | --- | --- |
|  | **Low-dose**  **(n=6)** | **Mid-dose**  **(n=6)** | **High-dose**  **(n=6)** | **Low-dose**  **(n=6)** | **Mid-dose**  **(n=6)** | **High-dose**  **(n=6)** | **Low-dose**  **(n=6)** | **Mid-dose**  **(n=6)** | **High-dose**  **(n=6)** | **Low-dose**  **(n=6)** | **Mid-dose**  **(n=6)** | **High-dose**  **(n=6)** | **Low-dose**  **(n=6)** | **Mid-dose**  **(n=6)** | **High-dose**  **(n=6)** | **Low-dose**  **(n=5)** | **Mid-dose**  **(n=5)** | **High-dose**  **(n=6)** |
| WBC  (10^9/^L) | 6.60±0.62 | 4.14±.033 | 6.10±0.64 | 6.67±0.72 | 4.20±0.37 | 6.20±0.67 | 6.704±0.46 | 4.30±0.40 | 5.88±0.42 | 7.17±0.62 | 3.86±0.32 | 5.87±0.62 | 7.28±0.72 | 4.36±0.36 | 6.92±0.69 | 7.56±0.72 | 4.35±0.39 | 7.19±0.20 |
| Neut  (10^9^/L) | 3.94±0.36 | 2.22±0.21 | 3.42±0.40 | 3.40±0.35 | 2.36±0.27 | 3.61±0.39 | 4.32±0.41 | 2.49±0.21 | 3.26±0.39 | 4.82±0.46 | 2.05±0.16 | 3.45±0.52 | 4.68±0.55 | 2.27±0.20 | 4.53±0.88 | 4.64±0.40 | 2.25±0.22 | 4.52±1.22 |
| Lymph  (10^9^/L) | 2.06±0.27 | 1.50±0.20 | 2.14±0.25 | 2.07±0.36 | 1.39±0.12 | 2.09±0.41 | 1.88±0.16 | 1.36±0.15 | 2.10±0.22 | 1.77±0.10 | 1.41±0.18 | 1.89±0.11 | 2.04±0.19 | 1.64±0.16 | 1.93±0.32 | 2.26±0.33 | 1.63±0.15 | 2.07±0.39 |
| Monocyte  (10^9^/L) | 0.50±0.07 | 0.32±0.05 | 0.40±0.07 | 0.50±0.08 | 0.33±0.05 | 0.40±0.07 | 0.44±0.06 | 0.32±0.05 | 0.40±0.06 | 0.48±0.08 | 0.31±0.04 | 0.41±0.06 | 0.47±0.05 | 0.33±0.03 | 0.40±0.04 | 0.58±0.10 | 0.33±0.04 | 0.46±0 |
| Eosinophil  (10^9^/L) | 0.09±0.03 | 0.09±0.02 | 0.11±0.03 | 0.07±0.01 | 0.10±0.02 | 0.08±0.02 | 0.09±0.02 | 0.10±0.02 | 0.11±0.03 | 0.08±0.02 | 0.08±0.02 | 0.10±0.03 | 0.08±0.02 | 0.11±0.02 | 0.05±0.01 | 0.07±0.02 | 0.10±0 | 0.12±0.07 |
| RBC  (10^12^/L) | 4.98±0.29 | 4.73±0.24 | 4.64±0.19 | 4.94±0.24 | 4.76±0.24 | 4.80±0.16 | 5.02±0.28 | 4.68±0.18 | 4.92±0.17 | 4.88±0.25 | 4.81±0.25 | 5.03±0.15 | 4.90±0.27 | 4.82±0.21 | 4.83±0.18 | 4.87±0.29 | 4.71±0.34 | 4.86±0.14 |
| HGB  (g/L) | 150.17±  9.17 | 144.67±  7.67 | 140.33±  6.05 | 149.00±  8.01 | 145.83±  7.53 | 144.83±  5.79 | 150.83±  8.67 | 142.67±  5.00 | 149.33±  5.27 | 146.67±  7.25 | 147.00±  8.21 | 153.20±  4.80 | 147.67±  8.26 | 148.00±  6.53 | 145.75±  5.85 | 150.00±  9.17 | 145.80±  10.30 | 148.17±  4.73 |
| PLT  (10^9^/L) | 247.83±  16.33 | 218.50±  12.41 | 234.67±  20.53 | 262.00±  18.12 | 225.17±  16.94 | 256.33±  23.88 | 240.33±  15.56 | 214.17±  16.86 | 250.33±  15.86 | 248.33±  11.55 | 223.67±16.14 | 254.80±  25.74 | 253.33±  22.35 | 230.00±  14.85 | 283.50±  30.80 | 264.00±  33.75 | 234.40±  14.45 | 258.17±  22.67 |
| Total T cells  (10^6^/L) | 1212.00±  91.03 | 937.67±  121.04 | 1417.67±  149.86 | 1336.17±  156.94 | 932.17±  92.88 | 1315.33±  309.76 | 1312.00±  136.94 | 860.00±  100.02 | 1405.20±  198.18 | 1282.33±  52.58 | 973.17±  145.19 | 1143.80±  115.93 | 1336.50±  161.51 | 1131.80±  125.54 | 1317.75±  226.19 | 1509.80±  252.92 | 1089.00±  166.14 | 1172.50±  237.64 |
| Total B cells  (10^6^/L) | 171.40±  14.48 | 134.33±  15.70 | 271.50±  47.30 | 182.67±  18.01 | 132.83±  18.04 | 218.00±  32.54 | 177.50±  19.26 | 128.67±  19.84 | 228.20±  23.50 | 206.67±  27.62 | 131.50±  15.53 | 228.80±  31.24 | 170.50±  13.27 | 147.00±  16.49 | 230.00±  24.11 | 221.60±  47.41 | 141.00±  18.67 | 216.17±  21.46 |
| NK cells  (10^6^/L) | 282.80±  110.29 | 325.17±  64.80 | 480.17±  139.26 | 242.67±  67.31 | 298.33±  35.47 | 494.83±  131.87 | 353.83±  98.91 | 261.00±  37.90 | 373.80±  90.58 | 288.33±  63.57 | 302.33±  38.08 | 408.00±  110.17 | 375.67±  111.61 | 367.20±  30.742 | 387.25±  72.47 | 429.00±  100.26 | 356.20±  12.70 | 395.50±  02.12 |
| Tregs (%) | 3.65±0.41 | 3.38±0.41 | 3.38±0.38 | 2.96±0.42 | 3.36±0.41 | 3.70±0.26 | 3.24±0.29 | 3.46±0.48 | 3.12±0.13 | 3.51±0.35 | 3.20±0.56 | 3.43±0.14 | 3.42±0.17 | 3.36±0.12 | 3.12±0.29 | 4.17±0.57 | 3.67±0.30 | 3.69±0.38 |
| Activated T cells(%) | 15.50±  2.03 | 18.90±  2.54 | 12.88±  1.68 | 15.66±  2.20 | 21.76±  2.97 | 14.67±  1.42 | 15.41±  1.37 | 20.61±  3.42 | 14.87±  2.19 | 16.35±  2.21 | 22.28±  3.57 | 13.88±  1.02 | 17.97±  1.78 | 23.71±  3.50 | 17.20±  1.22 | 17.31±  2.90 | 21.72±  3.46 | 13.06±  1.63 |
| CD3^+^CD4^+^ T cells (10^6^/L) | 775.20±  103.89 | 488.33±  95.82 | 847.50±  107.68 | 837.50±  108.07 | 538.67±  81.74 | 786.17±  180.82 | 801.00±  112.54 | 477.83±  69.42 | 767.40±  111.58 | 782.334±  5.95 | 531.33±  54.15 | 687.80±  87.20 | 793.67±  111.92 | 604.20±  87.07 | 763.00±  132.98 | 981.40±  229.27 | 568.40±  54.60 | 698.33±  130.34 |
| CD3^+^CD8^+^ T cells (10^6^/L) | 356.80±  41.68 | 345.17±  71.73 | 502.67±  74.27 | 423.00±  62.29 | 334.50±  51.34 | 450.33±  116.12 | 420.00±  44.54 | 319.50±  84.39 | 548.60±  89.57 | 417.00±  41.81 | 378.50±  113.95 | 392.80±  70.41 | 452.67±  61.06 | 436.60±  105.60 | 462.75±  78.15 | 431.20±  44.44 | 442.60±  145.52 | 396.17±  87.45 |
| CD4^+^/CD8^+^ | 2.37±0.56 | 1.86±0.48 | 1.94±0.38 | 2.18±0.38 | 1.89±0.50 | 2.02±0.39 | 2.04±0.35 | 1.98±0.49 | 1.55±0.31 | 2.02±0.29 | 2.00±0.52 | 2.07±0.50 | 1.89±0.31 | 1.81±0.55 | 1.71±0.31 | 2.32±0.51 | 1.76±0.48 | 2.03±0.37 |
| ALT  (U/L) | 29.83±10.71 | 19.33±5.90 | 18.33±8.67 | 32.50±11.50 | 23.17±6.25 | 29.33±14.44 | 21.67±5.55 | 23.33±3.97 | 24.17±11.26 | 19.17±2.60 | 18.50±3.39 | 20.60±7.52 | 27.00±6.96 | 16.33±2.60 | 12.75±3.71 | 23.00±2.94 | 18.60±5.66 | 23.50±7.87 |
| AST  (U/L) | 23.83±4.83 | 20.00±2.11 | 17.67±1.80 | 25.83±5.34 | 24.17±2.55 | 22.17±4.10 | 20.33±3.00 | 23.83±1.96 | 20.33±2.54 | 20.67±2.38 | 20.83±1.80 | 18.60±1.63 | 31.67±13.35 | 20.17±1.40 | 17.25±1.65 | 20.20±1.96 | 21.40±3.23 | 19.00±2.71 |
| ALB  (g/L) | 44.97±1.62 | 48.07±0.99 | 46.25±0.75 | 46.57±1.52 | 49.30±1.64 | 48.38±0.86 | 44.62±1.20 | 46.07±1.23 | 49.42±0.79 | 44.88±0.46 | 48.68±2.17 | 48.40±0.95 | 43.75±0.91 | 47.68±1.50 | 48.83±1.13 | 45.60±0.93 | 45.34±1.28 | 47.43±1.34 |
| GLB  (g/L) | 28.55±1.17 | 28.80±1.27 | 27.55±1.73 | 29.80±0.73 | 29.17±1.25 | 28.65±1.15 | 28.72±0.67 | 27.93±1.42 | 29.65±0.81 | 28.08±0.89 | 27.32±1.96 | 28.50±1.84 | 28.83±0.46 | 28.87±1.22 | 27.65±2.14 | 28.64±1.29 | 27.98±1.12 | 28.22±0.87 |
| TBIL  (μmol/L) | 10.03±1.28 | 11.72±2.36 | 9.37±1.14 | 8.78±0.68 | 9.55±1.73 | 8.88±1.48 | 10.13±2.12 | 11.30±3.15 | 12.17±1.38 | 10.28±1.84 | 10.52±2.07 | 11.18±1.05 | 8.13±1.40 | 11.17±2.34 | 12.25±2.53 | 10.56±1.17 | 9.96±2.28 | 8.75±1.20 |
| LDH  (U/L) | 180.00±13.93 | 190.17±20.60 | 183.33±21.72 | 187.17±8.99 | 183.00±12.43 | 181.00±14.93 | 173.17±9.86 | 199.17±20.17 | 186.17±10.49 | 180.67±14.31 | 196.83±7.37 | 197.40±20.49 | 196.00±17.14 | 203.33±26.00 | 171.50±13.58 | 181.20±14.77 | 181.80±10.91 | 179.83±19.71 |
| ALP  (U/L) | 62.17±3.12 | 61.67±4.20 | 57.67±4.49 | 64.50±3.59 | 61.50±4.70 | 61.33±5.09 | 64.33±1.99 | 60.50±4.44 | 61.50±5.27 | 62.17±3.30 | 61.83±4.21 | 61.00±4.81 | 66.33±2.50 | 65.50±4.95 | 56.25±5.12 | 72.00±3.29 | 69.20±7.29 | 60.67±4.20 |
| CK  (U/L) | 97.33±17.35 | 92.33±10.60 | 105.17±27.35 | 319.17±231.68 | 81.17±9.44 | 85.83±11.88 | 102.33±18.86 | 119.00±46.99 | 85.67±9.49 | 174.00±79.68 | 79.33±11.94 | 90.60±8.58 | 94.20±19.21 | 86.83±11.34 | 87.00±9.83 | 91.40±26.93 | 124.80±44.24 | 93.83±9.58 |
| GLU  (mmol/L) | 5.19±0.17 | 5.02±0.10 | 5.01±0.21 | 4.87±0.24 | 5.08±0.11 | 5.12±0.13 | 5.05±0.12 | 4.89±0.09 | 5.50±0.57 | 4.93±0.14 | 4.91±0.14 | 4.97±0.23 | 4.93±0.13 | 5.07±0.07 | 4.91±0.22 | 5.12±0.24 | 5.11±0.11 | 5.34±0.14 |
| TG  (mmol/L) | 1.34±0.26 | 1.00±0.06 | 1.50±0.13 | 0.97±0.26 | 1.44±0.21 | 1.36±0.22 | 1.39±0.20 | 1.31±0.23 | 1.39±0.29 | 1.16±0.28 | 1.27±0.10 | 1.64±0.24 | 1.27±0.32 | 1.33±0.12 | 1.62±0.28 | 1.49±0.29 | 1.27±0.19 | 1.68±0.20 |
| CHOL  (mmol/L) | 4.40±0.36 | 4.59±0.25 | 4.47±0.50 | 4.28±0.35 | 4.76±0.41 | 4.56±0.37 | 4.29±0.29 | 4.70±0.34 | 4.72±0.47 | 4.06±0.30 | 4.63±0.32 | 5.01±0.47 | 3.74±0.30 | 4.81±0.31 | 4.31±0.52 | 4.40±0.43 | 4.79±0.36 | 4.83±0.46 |
| UREA  (mmol/L) | 4.84±0.47 | 5.51±0.63 | 4.38±0.45 | 4.80±0.46 | 5.12±0.52 | 4.43±0.75 | 4.48±0.25 | 4.99±0.59 | 4.78±0.79 | 4.89±0.23 | 4.78±0.44 | 3.60±0.35 | 5.17±0.66 | 4.76±0.26 | 4.09±0.64 | 4.70±0.42 | 5.26±0.43 | 4.17±0.38 |
| Cr  (μmol/L) | 69.50±7.26 | 75.50±7.95 | 76.83±5.64 | 73.17±7.36 | 74.50±8.35 | 72.83±6.74 | 69.83±6.34 | 74.67±7.42 | 73.17±7.38 | 69.00±6.18 | 73.83±7.50 | 74.60±6.59 | 69.83±6.71 | 73.83±7.01 | 73.25±6.69 | 67.40±8.16 | 69.20±8.45 | 72.83±5.74 |
| K^+^  (mmol/L) | 4.07±0.19 | 4.27±0.08 | 4.28±0.17 | 4.19±0.11 | 4.27±0.08 | 4.16±0.06 | 4.16±0.19 | 4.19±0.11 | 4.32±0.09 | 3.99±0.09 | 4.37±0.14 | 4.34±0.19 | 4.17±0.10 | 4.13±0.15 | 4.04±0.17 | 3.76±0.13 | 4.12±0.11 | 4.22±0.09 |
| Na^+^  (mmol/L) | 141.12±0.79 | 139.80±0.60 | 141.13±0.89 | 140.78±0.93 | 140.02±0.49 | 141.30±0.62 | 140.72±0.29 | 139.95±0.60 | 140.75±0.47 | 141.02±0.71 | 139.45±0.63 | 140.48±0.86 | 139.75±0.75 | 139.70±0.39 | 140.98±0.41 | 140.82±0.87 | 139.58±0.40 | 140.42±0.30 |
| Cl^-^  (mmol/L) | 102.35±1.01 | 101.80±0.69 | 102.73±0.63 | 101.72±0.81 | 101.83±1.13 | 102.65±0.31 | 102.35±0.38 | 101.38±0.91 | 101.68±0.61 | 101.93±1.09 | 101.87±0.85 | 102.74±0.68 | 102.22±1.19 | 102.68±0.31 | 103.40±0.81 | 102.04±1.08 | 102.50±0.58 | 102.63±0.37 |
| Ca^2+^  (mmol/L) | 2.32±0.06 | 2.36±0.04 | 2.35±0.02 | 2.36±0.06 | 2.41±0.05 | 2.38±0.02 | 2.29±0.04 | 2.37±0.03 | 2.41±0.02 | 2.32±0.04 | 2.39±0.07 | 2.38±0.05 | 2.32±0.04 | 2.34±0.03 | 2.38±0.05 | 2.28±0.05 | 2.32±0.05 | 2.35±0.03 |
| Mg^2+^  (mmol/L) | 0.88±0.03 | 0.90±0.02 | 0.87±0.01 | 0.90±0.03 | 0.90±0.03 | 0.87±0.01 | 0.88±0.02 | 0.89±0.02 | 0.92±0.02 | 0.87±0.03 | 0.88±0.01 | 0.92±0.02 | 0.87±0.03 | 0.89±0.01 | 0.87±0.01 | 0.90±0.03 | 0.88±0.04 | 0.91±0.01 |
| PT (s) | 13.17±0.41 | 13.17±0.37 | 12.82±0.21 | 13.23±0.40 | 13.13±0.23 | 12.68±0.14 | 13.35±0.42 | 13.35±0.46 | 12.65±0.17 | 13.15±0.38 | 13.28±0.36 | 12.32±0.16 | 13.25±0.29 | 13.17±0.41 | 12.78±0.26 | 12.64±0.31 | 12.86±0.32 | 12.78±0.13 |
| APTT (s) | 35.52±1.21 | 40.58±2.67 | 38.92±1.92 | 37.02±1.50 | 40.75±2.25 | 39.35±1.80 | 37.17±1.64 | 40.38±2.11 | 39.60±1.39 | 36.63±1.35 | 40.60±2.46 | 38.70±1.55 | 36.72±1.55 | 40.75±1.63 | 39.08±2.42 | 35.34±1.18 | 40.08±3.32 | 37.82±1.81 |
| TT (s) | 17.17±0.36 | 16.87±0.24 | 16.63±0.33 | 16.85±0.40 | 16.20±0.24 | 16.15±0.33 | 17.15±0.33 | 17.05±0.32 | 16.08±0.32 | 17.02±0.32 | 17.00±0.32 | 15.96±0.15 | 16.90±0.18 | 16.75±0.38 | 16.38±0.26 | 17.26±0.33 | 17.12±0.29 | 16.85±0.21 |
| FIB (g/L) | 2.88±0.15 | 3.23±0.36 | 2.73±0.13 | 3.22±0.22 | 3.43±0.18 | 3.06±0.25 | 3.17±0.22 | 3.08±0.20 | 2.92±0.14 | 3.06±0.24 | 3.01±0.25 | 2.97±0.24 | 3.31±0.22 | 3.03±0.18 | 2.65±0.27 | 2.90±0.20 | 2.86±0.31 | 2.90±0.24 |
| IL-1β  (pg/mL) | 5.00±0 | 5.00±0 | 5.13±0.13 | 5.00±0 | 5.80±0.80 | 5.48±0.48 | 5.00±0 | 6.28±1.28 | 5.00±0 | 5.00±0 | 5.65±0.65 | 5.00±0 | 5.00±0 | 6.67±1.67 | 5.30±0.19 | 5.00±0 | 8.82±3.82 | 7.07±1.42 |
| IL-2  (U/mL) | 320.33±45.27 | 234.33±33.26 | 270.17±29.62 | 302.83±51.84 | 246.17±39.46 | 286.17±27.78 | 309.33±63.34 | 236.50±27.63 | 280.33±26.90 | 244.50±37.95 | 251.67±18.92 | 282.83±35.47 | 308.50±55.71 | 240.17±21.98 | 299.33±31.92 | 270.80±60.34 | 254.80±32.12 | 338.00±42.19 |
| IL-6  (pg/mL) | 2.08±0.40 | 3.01±0.76 | 1.61±0.10 | 1.79±0.24 | 2.07±0.30 | 1.89±0.25 | 2.53±0.61 | 5.28±2.97 | 2.49±0.99 | 4.16±1.50 | 1.69±0.12 | 1.54±0.04 | 5.05±2.60 | 1.71±0.13 | 1.81±0.30 | 4.55±1.94 | 3.76±1.47 | 2.52±0.62 |
| IL-10  (pg/mL) | 5.03±0.03 | 5.00±0 | 5.00±0 | 5.00±0 | 5.00±0 | 5.00±0 | 5.13±0.13 | 5.00±0 | 5.03±0.03 | 5.00±0 | 5.12±0.12 | 5.00±0 | 5.00±0 | 5.03±0.03 | 5.25±0.25 | 5.00±0 | 5.00±0 | 5.00±0 |
| ESR  (mm/H) | 3.67±1.12 | 5.00±1.92 | 3.83±0.79 | 3.67±0.92 | 7.50±3.98 | 6.00±1.46 | 4.83±1.42 | 4.67±1.99 | 4.00±1.00 | 3.00±0.82 | 5.33±2.17 | 3.83±0.79 | 3.50±0.62 | 4.67±1.78 | 4.33±0.84 | 3.40±0.68 | 4.60±2.36 | 4.33±0.96 |
| TNF-α  (pg/mL) | 5.75±1.07 | 6.33±2.23 | 6.08±0.74 | 4.65±0.23 | 6.17±1.16 | 6.17±0.66 | 4.70±0.45 | 6.17±0.81 | 6.50±1.51 | 4.00±0 | 5.15±0.33 | 5.67±0.62 | 12.75±8.21 | 5.55±0.34 | 7.80±2.26 | 11.04±2.98 | 11.02±4.70 | 5.72±0.41 |
| Hs-CRP  (mg/L) | 1.10±0.18 | 1.48±0.99 | 0.68±0.17 | 1.72±0.63 | 0.77±0.21 | 2.90±2.36 | 2.15±0.84 | 0.65±0.18 | 0.83±0.18 | 2.63±1.92 | 0.68±0.22 | 0.57±0.12 | 1.05±0.27 | 0.65±0.13 | 0.50±0.15 | 2.74±1.47 | 0.70±0.24 | 0.98±0.28 |

Abbreviations: WBC, white blood cells; RBC, [red blood cell](javascript:;)s; HGB, [hemoglobin](javascript:;); PLT, platelet; ALT, alanine aminotransferase; AST, aspartate aminotransferase; TP, total protein; ALB, albumin; GLB, globulin; TBIL, total bilirubin; LDH, [lactic dehydrogenase](javascript:;); ALP, [alkaline phosphatase](javascript:;); CK, [creatine kinase](javascript:;); GLU, glucose; TG, triglyceride; CHOL, cholesterol; Cr, creatinine; PT, [prothrombin time](javascript:;); APTT, activated partial thromboplastin time. TT, [thrombin time](javascript:;); FIB, fibrinogen. IL, [interleukin](javascript:;); ESR, [erythrocyte sedimentation rate](javascript:;); TNF, tumor necrosis factor; Hs-CRP, high sensitivity C-reactive protein; Data are presented as mean ± SEM.

**Table S9.** **The reference range and normal value of blood test.**

| **Variables** | **Reference range** | **Healthy person 1** | **Healthy person 2** | **Healthy**  **person 3** | **Healthy**  **person 4** | **Healthy**  **person 5** |
| --- | --- | --- | --- | --- | --- | --- |
| WBC (10^9/^L) | 3.5-9.5 | 6.44 | 6.51 | 4.52 | 5.04 | 4.56 |
| Neut (10^9^/L) | 1.8-6.3 | 3.59 | 3.95 | 2.54 | 3.24 | 2.08 |
| Lymph (10^9^/L) | 1.1-3.2 | 2.42 | 2.00 | 1.60 | 1.57 | 2.14 |
| Monocyte (10^9^/L) | 0.1-0.6 | 0.29 | 0.50 | 0.32 | 0.19 | 0.26 |
| Eosinophil (10^9^/L) | 0.02-0.52 | 0.10 | 0.05 | 0.05 | 0.03 | 0.06 |
| RBC (10^12^/L) | 3.8-5.1 (female)  4.3-5.8 (male) | 5.04 | 5.02 | 4.03 | 4.78 | 5.53 |
| HGB (g/L) | 115.0-150.0 (female)  130.0-175.0 (male) | 157.0 | 154.0 | 122.0 | 151.0 | 160.0 |
| PLT (10^9^/L) | 125.0-350.0 | 173.0 | 188.0 | 194.0 | 208.0 | 285.0 |
| Total T cells (10^6^/L) | 955.0-2860.0 | 2053.0 | 1226.0 | 1386.0 | 1299.0 | 1667.0 |
| Total B cells (10^6^/L) | 90.0-560.0 | 172.0 | 210.0 | 232.0 | 241.0 | 241.0 |
| NK cells (10^6^/L) | 150.0-1100.0 | 276.0 | 578.0 | 305.0 | 204.0 | 436.0 |
| Tregs (%) | 3.1-6.5 | 3.1 | 3.5 | 4.9 | 5.1 | 4.7 |
| Activated T cells (%) | 9.0-25.6 | 14.2 | 17.1 | 19.2 | 24.9 | 15.2 |
| CD3^+^CD4^+^ T cells (10^6^/L) | 550.0-1440.0 | 1086.0 | 614.0 | 840.0 | 803.0 | 1119.0 |
| CD3^+^CD8^+^ T cells (10^6^/L) | 320.0-1250.0 | 916.0 | 487.0 | 472.0 | 402.0 | 474.0 |
| CD4^+^/CD8^+^ (ratio) | 0.71-2.78 | 1.19 | 1.26 | 1.78 | 2.00 | 2.36 |
| ALT (U/L) | <42 | 11 | 12 | 10 | 15 | 16 |
| AST (U/L) | <41 | 22 | 21 | 15 | 22 | 19 |
| ALB (g/L) | 35.0-52.0 | 51.3 | 50.9 | 47.4 | 46.3 | 47.2 |
| GLB (g/L) | 20.0-35.0 | 25.6 | 28.2 | 31.4 | 29.2 | 32.4 |
| TBIL (μmol/L) | <26 | 10.7 | 14.8 | 19.5 | 13.0 | 7.4 |
| LDH (U/L) | 135-214 | 175 | 188 | 172 | 160 | 160 |
| ALP (U/L) | 35-105 | 58 | 53 | 83 | 55 | 82 |
| CK (U/L) | <171 | 152 | 90 | 17 | 136 | 51 |
| GLU (mmol/L) | 4.1-6.1 | 5.21 | 5.14 | 4.34 | 4.94 | 4.94 |
| TG (mmol/L) | <1.7 | 0.69 | 0.64 | 0.65 | 0.78 | 1.24 |
| CHOL (mmol/L) | <5.2 | 3.96 | 3.65 | 4.30 | 4.99 | 4.57 |
| UREA (mmol/L) | 2.6-8.0 | 8.0 | 7.5 | 4.3 | 5.4 | 5.1 |
| Cr (μmol/L) | 59-104 | 93 | 92 | 57 | 62 | 89 |
| K^+^ (mmol/L) | 3.5-5.1 | 4.2 | 4.2 | 4.0 | 4.4 | 4.1 |
| Na^+^ (mmol/L) | 136.0-145.0 | 141.8 | 137.5 | 142.8 | 138.0 | 137.6 |
| Cl^-^ (mmol/L) | 99.0-110.0 | 108.4 | 101.7 | 105.0 | 103.5 | 102.6 |
| Ca^2+^ (mmol/L) | 2.2-2.5 | 2.3 | 2.3 | 2.4 | 2.3 | 2.4 |
| Mg^2+^ (mmol/L) | 0.7-1.1 | 0.8 | 0.9 | 0.7 | 0.8 | 0.9 |
| PT (s) | 9.4-12.5 | 12.7 | 12.4 | 11.5 | 10.4 | 11.0 |
| APTT (s) | 25.1-42.0 | 34.4 | 33.0 | 27.7 | 38.0 | 28.5 |
| TT (s) | 14.0-19.0 | 15.6 | 15.4 | 14.0 | 14.4 | 14.5 |
| FIB (g/L) | 2.4-5.0 | 2.8 | 3.0 | 4.6 | 3.1 | 3.1 |
| IL-1β (pg/mL) | <6 | 5 | 5 | 5 | 5 | 5 |
| IL-2R (U/mL) | 223-710 | 243 | 327 | 442 | 233 | 386 |
| IL-6 (pg/mL) | <7.0 | 1.5 | 3.8 | 1.5 | 1.5 | 2.4 |
| IL-10 (pg/mL) | <9.1 | 5.0 | 5.0 | 5.7 | 5.0 | 5.2 |
| ESR (mm/H) | 0-20.0 | 2.0 | 10.0 | 6.0 | 2.0 | 5.0 |
| TNF-α (pg/mL) | <8.1 | 5.0 | 5.0 | 5.0 | 5.0 | 5.0 |
| Hs-CRP (mg/L) | <1.0 | 0.4 | 0.8 | 0.7 | 0.2 | 0.1 |

Abbreviations: WBC, white blood cells; RBC, red blood cells; HGB, hemoglobin; PLT, platelet; ALT, alanine aminotransferase; AST, aspartate aminotransferase; TP, total protein; ALB, albumin; GLB, globulin; TBIL, total bilirubin; LDH, lactic dehydrogenase; ALP, alkaline phosphatase; CK, creatine kinase; GLU, glucose; TG, triglyceride; CHOL, cholesterol; Cr, creatinine; PT, prothrombin time; APTT, activated partial thromboplastin time. TT, thrombin time; FIB, fibrinogen. IL, interleukin; ESR, erythrocyte sedimentation rate; TNF, tumor necrosis factor; Hs-CRP, high sensitivity C-reactive protein.

**Table S10. Knee MRI scan protocol acquisition parameters.**

| **Scan** | **Localizer** | **COR T1W** | **COR**  **T2** | **COR**  **PD** | **SAG**  **T2** | **SAG**  **PD** | **TRA**  **T2** |
| --- | --- | --- | --- | --- | --- | --- | --- |
| Plane | 3-plane | Coronal | Coronal | Coronal | Sagittal | Sagittal | Traverse |
| Matrix (phase) | 128 | 224 | 224 | 224 | 224 | 224 | 224 |
| Matrix (frequency) | 256 | 320 | 320 | 320 | 320 | 320 | 320 |
| No. of slices | 10 | 20 | 20 | 20 | 24 | 24 | 20 |
| FOV (mm) | 300 | 140 | 140 | 140 | 140 | 140 | 170 |
| Slice thickness (mm) | 9/0 | 3/0 | 3/0 | 3/0 | 3/0 | 3/0 | 3/0 |
| TE/TR | 5/10 | 12/480 | 80/2200 | 20/3200 | 80/2200 | 20/3200 | 80/3600 |
| Bandwidth (Hz/pixel) | 250 | 83.3 | 50 | 50 | 50 | 50 | 62.5 |
| ETL | 1 | 3 | 16 | 16 | 16 | 16 | 18 |
| Phase encode axis | A/P, R/L | R/L | R/L | R/L | A/P | A/P | R/L |
| Phase resolution | 50 | 100 | 100 | 100 | 100 | 100 | 100 |
| X-resolution | 0.39 | 0.44 | 0.44 | 0.44 | 0.44 | 0.44 | 0.44 |
| Y-resolution | 0.78 | 0.63 | 0.63 | 0.63 | 0.63 | 0.63 | 0.63 |

Abbreviations: COR, coronal; SAG, sagittal; TRA, traverse; T1W, T1 Weighted Imaging; T2, T2 Weighted Imaging; PD, proton density; FOV, field of view; TE, echo time; TR, repetition time; ETL, echo-train length. Parameters will be slightly fine-tune according to patient’s weight and height.
